# Competitive LC-MS/MS assay to investigate protein metalation dynamics

**DOI:** 10.1039/d5qi02457a

**Published:** 2026-02-19

**Authors:** Kira Küssner, Michael Wolf, Andrea Cucchiaro, Christian G. Hartinger, Samuel Meier-Menches, Monika Cziferszky

**Affiliations:** a Institute for Pharmacy, Pharmaceutical Chemistry, Department of Chemistry and Pharmacy, Center for Molecular Bioscience (CMBI), University of Innsbruck Innrain 80/82 A-6020 Innsbruck Austria monika.cziferszky@uibk.ac.at; b Institute of Analytical Chemistry, Faculty of Chemistry, University of Vienna Waehringer Str. 38 A-1090 Vienna Austria samuel.meier-menches@univie.ac.at; c Doctoral School in Chemistry, University of Vienna Waehringer Str. 38 A-1090 Vienna Austria; d Faculty of Science, School of Chemical Science, University of Auckland Science Centre 302 – Bldg 302 23 Symonds St Auckland Central 1010 Auckland New Zealand; e Institute of Inorganic Chemistry, Faculty of Chemistry, University of Vienna Waehringer Str. 42 A-1090 Vienna Austria; f Joint Metabolome Facility, Medical University of Vienna and University of Vienna Waehringer Str. 38 A-1090 Vienna Austria

## Abstract

Knowledge gaps in the biospeciation of potential metallodrugs may lead to the generalized assumption of their promiscuous reactivity and inherent toxicity, neglecting their pharmaceutical potential. Herein, we developed a rapid and competitive LC-MS/MS assay to determine metalation dynamics of protein mixtures by metallodrugs. Specifically, the time-dependent reactivity of different metallodrugs, based on platinum(ii) (cisplatin, [Pt(ala)(ASA-But)Cl]), ruthenium(ii) ([Ru(HQ)(Cym)Cl], [Ru(NHC)(Cym)Cl_2_]), and iridium(iii) ([Ir(HQ)(Cp*)Cl], [Ir(NHC)(Cp*)Cl_2_]), towards an equimolar protein mixture was investigated. The assay revealed metal-dependent selectivity of adduct formation and subsequent deactivation by cellular detoxifying nucleophiles, *e.g.* glutathione (GSH). Online top-down fragmentation further enabled the localization of binding sites of metallodrugs on proteins in the same run, which could be directly related to complex speciation behaviour. The reactivity of Zeise's salt derivative [Pt(ala)(ASA-But)Cl] (ala = l-alanine; ASA-But = but-3-en-1-yl 2-acetoxybenzoate) with sulfur donor atoms was found to exceed all investigated compounds, including cisplatin. Ruthenium compounds preferred N-donor coordination, which resulted in a strong affinity towards histidine residues. Protein adducts of the somewhat redox-active iridium compounds were quickly transformed into stable adducts with detoxifying nucleophiles, especially with GSH. This model system provides further evidence that metallodrug reactivity is more selective in competitive settings, as would be anticipated from exposure to single biomolecules. This implies that the intracellular selectivity of metallodrugs can be chemically tuned and that this aspect may be accounted for in future metallodrug design. Such design strategies will be supported by expanding the present competitive assay to more complex systems that better mimic physiological intra- and extracellular tumour microenvironments.

## Abstract 2

Speciation analysis reveals selective binding in competitive assays. Metal-dependent binding selectivities may be exploited to guide future metallodrug design.

## Graphical abstract

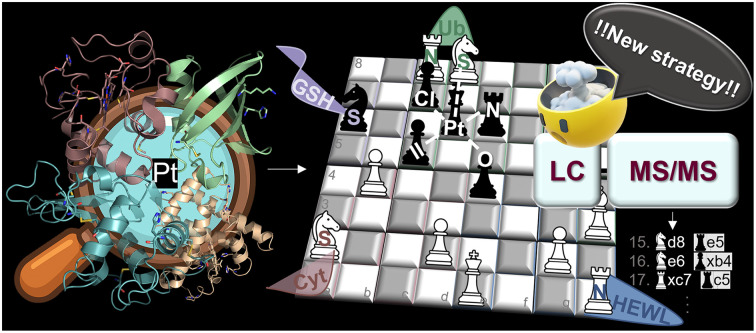

## Introduction

Biologically-active metal complexes are unique due to their high reactivity towards biomolecules^1^ and potentially offer a wide range of pharmaceutical opportunities, which are often complementary to the scope of organic molecules.^2,3^ Nevertheless, the high reactivity also fuels the prejudice of their unspecific modes-of-action and toxicity. Many studies investigating metal–protein interactions indicate selectivity of metal compounds, *e.g.* by (selective) activation^4–7^ or interaction with specific sites on protein surfaces,^8,9^ depending on the ligand coordination sphere and the chemical environment of the coordinating biomolecule. Common cellular downstream effects of metallodrugs are DNA damage, ROS generation, disturbance of the redox balance, and mitochondrial dysfunction, among others.^3,6^ This outlook suggests the possibility for smart ligand engineering with improved target specificity of metal compounds, based on a fundamental understanding of molecular metalation and speciation dynamics.

Proteins are increasingly considered to play a pivotal role in speciation and the modes-of-action of metal-based drugs, not only being involved in transportation pathways, drug activation/deactivation, and cellular uptake, but also acting as main targets.^10,11^ However, better and more sophisticated model systems are required to understand and exploit these molecular interactions for pharmacological benefits.^12–14^ Additionally, the fate of metallodrugs in a cellular environment is often dependent on their exchange and speciation behaviour with highly abundant small nucleophiles, such as GSH and amino acids. Reactivity towards GSH represents detoxification and deactivation processes and thus, influences the intracellular concentrations of metallodrugs.^15–17^

The investigation of protein metalation dynamics and the in-solution equilibria of ligand exchange rates remains challenging,^18,19^ as metal complexes exhibit a highly diverse reactivity profile towards proteins and the microenvironment of the binding site. To date, there is no reliable one-fits-all screening method for a comprehensive characterization of this reactivity profile. Many mass spectrometry (MS)-based methods were explored to determine stoichiometry, binding sites, speciation, and stability in direct interactions of the drugs with smaller biomolecular model systems.^9,17,20–26^ For example, a hyphenated capillary electrophoresis MS approach was used to identify preferences of metallodrugs to interact with DNA or proteins,^27^ however, CE–MS systems are not widely available and methods are not simple to standardize.

Despite the large body of MS-based investigations to assess the reactivity and binding selectivity of metal complexes to proteins, only a limited number of studies have employed hyphenation of liquid chromatography (LC) and top-down mass spectrometry.^28^ To explore the reactivity and potential selectivity rather than promiscuity, we established a competitive top-down LC-MS/MS assay, suitable for a broad range of metal compounds and model proteins. We were able to gain important information about adduct stoichiometry, affinities, binding site selectivity, and metal complex speciation upon coordination, as well as the biologically relevant reversibility of the metalation process when exposed to detoxifying agents.

## Experimental

### Reagents

Proteins and nucleophiles were purchased from Sigma-Aldrich (cytochrome c (Cyt) from equine heart; ubiquitin (Ub) from bovine erythrocytes; hen egg white lysozyme (HEWL); myoglobin (Myo) from equine heart; GSH; and methionine (Met)). Cisplatin (*cis*-diaminodichloroplatinum(ii) – compound 1) was purchased from BLD pharm and used as received. Compound 2 ^29^ and compounds 3–6 ^30,31^ were synthesized according to literature procedures. Stock solutions of the proteins were prepared in Milli-Q water.

### Incubations

Stock solutions of the metal complexes (2 mM in H_2_O (1), or MeOH (2–6)) were prepared and added to the aqueous solution of the protein mixture to yield a 5 : 1 : 1 : 1 : 1 ratio (M : Ub : Cyt : HEWL : Myo) (M = compound 1–6) at 20 °C. For nucleophile exchange experiments the excess complex was filtered off using a Nanosep filter (Amicon Ultra – 0.5 mL 3 K, 2 × 5 min at 10 000 rcf) and 8 eq. GSH + Met were added. The incubation mixtures were analysed every two hours (2–24 h) before nucleophile exposure and 2 h and 24 h after adding GSH + Met. Precisely timed blank methods stalled the instrument for the desired measuring time points. Autosampler temperature was set to 20 °C and each sample was measured three times.

### Incubations in buffer

50 µM stock solutions of the proteins were prepared in 20 mM TEAB (pH = 8) and incubated with the 2 mM stock solution of the complexes 3 and 4 in MeOH with a final ratio of (5 : 1 : 1 : 1 : 1) (Metal : Cyt : Ub : HEWL : Myo). TEAB was added to yield a final concentration of 10 µM of each protein. LC-MS measurements were conducted after 24 h incubation at 20 °C. The excess compound was removed by centrifugation using vivaspin 500 (3000 MWCO PES) at RT at 15 000*g* in a fixed angle centrifuge. A 400 µM nucleophile mix (GSH + Met) stock solution was prepared in TEAB and eight equivalents were added to the incubation mixture. LC-MS measurements were conducted after 2 h and 24 h.

### Analysis

All mixtures were chromatographically separated using a Vanquish UHPLC System (Thermo Fisher Scientific) with a Poroshell 300SB-C8 Column (1.0 × 75 mm, 5 µm) and analysed on a Q Exactive HF (Thermo Fisher Scientific) mass spectrometer. Mobile phases consisted of H_2_O/FA (100/0.2; v/v) (A) and ACN/MeOH/FA (90/10/0.2; v/v/v) (B). The chromatographic gradient was set to 10–40% B in 2.5 min, 40–50% B in 0.2 min, 50–95% B in 0.1 min, column wash with 95% B for 0.5 min, 95–10% B in 0.1 min, and re-equilibration with 10% B for 1.1 min, with a total run time of 4.5 min. The flow rate was set to 0.6 mL min^−1^ with an injection volume of 2 µL and a column temperature of 50 °C. A solution of MeOH/H_2_O/FA (50/50/0.2; v/v/v) was used as needle wash.

Electrospray ionization (ESI) mass spectra of the reaction mixtures were recorded on a Q Exactive HF (Thermo Fisher Scientific) in positive ion mode. Samples were ionized using a HESI-II source with a spray voltage of 3.5 kV and the following parameters: transfer tube: 320 °C, sheath gas: 52.5 a.u.^−1^, aux gas: 13.75 a.u.^−1^, sweep gas: 2.75 a.u.^−1^, aux gas heater 350 °C, and S lens RF level: 50%. The scan range was set to *m*/*z* 350–2000 on MS^1^ level and *m*/*z* 150–2000 on MS/MS level.

For discovery of precursor ions, the resolution was set to 120 000 at *m*/*z* 200, the maximum injection time to 500 ms and the AGC-target to 5e^6^ for a MS^1^ only method. Due to numerous protein isotopologues, the risk of space-charge effects was deemed negligible, justifying the use of high AGC-targets. Specific inclusion lists of possible reaction products for each compound were compiled from the data generated with this method.

For the top-down experiments a data dependent Top 1 method with the respective inclusion list for each compound was applied. Here, the resolution, maximum injection time, and AGC-target were set to 4.5e^4^, 246 ms, and 5e^6^ for MS^1^ level and 60 000, 246 ms, and 5e^5^ for MS/MS level, respectively. The isolation window was set to 3.0 *m*/*z* and a minimum intensity threshold of 3.3e^4^ was used. Samples were injected multiple times using different normalized collision energies (NCEs) of 20, 25, 30, 40, or 50.

### Data evaluation

Data analysis was performed using the Xcalibur software package (Thermo Fisher Scientific), which also controlled the experimental setup, and the Apm^2^s software tool^32^ for fragment identification. Used isotopologues and sum formulae of proteins and complexes are listed in Table S1.

## Results and discussion

**Scheme 1:**
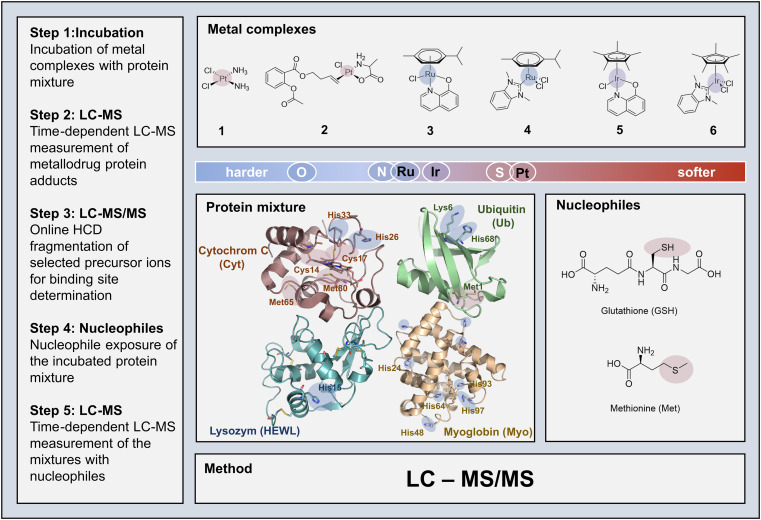
Competitive LC-MS/MS experimental approach for different metal complexes (compounds 1–6) with model proteins and small nucleophiles. Potential binding sites and metal centres highlighted with softer (red) and harder (blue) character.

In order to establish the envisaged LC-MS/MS platform for metallodrug analysis, we selected model proteins and metal complexes with different reactivity profiles: cytochrome c (Cyt), ubiquitin (Ub), hen egg white lysozyme (HEWL), myoglobin (Myo), 1 – [*cis*-(Pt(NH_3_)_2_(Cl)_2_)], 2 – [Pt(ala)(ASA-But)Cl], 3 – [Ru(HQ)(Cym)Cl], 4 – [Ru(NHC)(Cym)Cl_2_], 5 – [Ir(HQ)(Cp*)Cl], and 6 – [Ir(NHC)(Cp*)Cl_2_] (with ala = l-alanine, ASA-But = acetylsalicylicacid-butenyl, HQ = 8-oxyquinolinato, Cym = *p*-cymene, NHC = N-heterocyclic carbene, Cp* = pentamethylcyclopentadienyl). For a schematic overview of the competitive experimental LC-MS/MS approach see Scheme 1. Since LC-MS systems capable of top-down fragmentation techniques (MS/MS)^33^ are widespread in many laboratories nowadays, the presented method may be implemented in the general toolbox for selectivity investigations of metallodrugs.

The hard soft acid base (HSAB) theory can serve as a primary guideline for a generalized prediction of binding affinities (Scheme 1). However, the propensity of protein adduct formation and speciation characteristics of complexes is influenced by a variety of other factors too. These include mainly ligand exchange reactivity and in-solution equilibria governed by the *trans*-effect (in square planar Pt-complexes), hapticity of ligands, as well as the inertness and redox behaviour of the metal centre. Model proteins Ub, Cyt, HEWL and Myo were chosen based on two aspects: firstly, their molecular weight falls within the limit of top-down MS detection; and secondly, they provide characteristic reactivity profiles (*e.g.* mono-/bidentate binding motifs, spatial constraints, different donor atoms, presence/absence of heme, *etc*.), which can be used to systematically track the metallodrug behaviour when exposed to different chemical environments. Despite being the smallest of the four proteins, Ub is a suitable model protein to study the binding selectivity, as it provides a potential bidentate binding site on the N-terminal Met with a [M(S_Met1_,N_Met1_)] binding motif, a surface exposed nitrogen donor (N^ε^_His68_), and several accessible oxygen donors (O^δ1^_Asp21,39,52,58_). The structure of Cyt provides three accessible thioethers (S_Met65_, S_Met80_, and S_Cys17_) and two N-donors (N^ε^_His26_ and N^ε^_His33_) on the surface of the protein. N^ε^_His15_ and, to a lesser extent N_Lys33_ and O^δ1^_Asp101_, were reported as metalation sites in previous studies in HEWL, when exposed to an excess of the metal compounds.^9,34^ Myo features several histidine (His) residues and a less accessible Met residue. An overview of the protein sequences with potential binding sites highlighted is presented in Table S2. The competitive setting of the experiment allowed for evaluating specific reactivities of the different compounds for specific proteins and donor atoms, while the comparison between the different metal complexes provided insights into the influence of the ligand coordination sphere.

### Reactivity studies and complex speciation

**Fig. 1:**
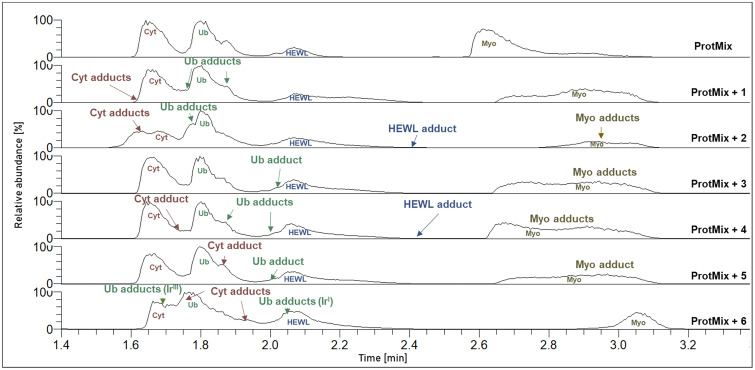
TICs after 24 h incubation of the protein mix and the protein-compound mixtures (1 – [Pt^II^(NH_3_)_2_Cl_2_]; 2 – [Pt^II^(ala)(ASA-But)Cl]; 3 – [Ru^II^(HQ)(cym)Cl]; 4 – [Ru^II^(NHC)(cym)Cl_2_]; 5 – [Ir^III^(HQ)(Cp*)Cl]; 6 – [Ir^III^(NHC)(Cp*)Cl_2_]). Identified adducts are indicated at their respective retention times.

Fig. 1 features the total ion chromatograms (TICs) after 24 h incubation of compounds 1–6 with the protein mixture in aqueous solution at 20 °C, with proteins and protein adducts indicated at their respective retention times (RT; for detailed information of the metalated adducts see Fig. S1 and Table S3). Adduct formation was observed with all metal complexes in different intensities and stoichiometries. Identification of reaction products was conducted by interpretation of the respective full mass spectra and targeted fragment spectra derived from LC-MS/MS analysis.

#### Platinum compounds (1 + 2)

Compound 1 formed low-abundance Cyt and Ub adducts with the main adduct composition being [prot + Pt(NH_3_)_2_Cl] (prot = Cyt, Ub) for both proteins. With Cyt we observed the loss of the NH_3_ ligands in lower abundant adducts [Cyt + Pt(NH_3_)_*n*_Cl] (*n* = 0, 1), while in Ub the loss of chlorido ligands is displayed by the adduct [Ub + Pt(NH_3_)_2_] (Fig. S1 and Table S3), as also found in previous top-down MS studies.^35^ The results indicate that organoplatinum compound 2 exhibited a notably higher reactivity than the other compounds, in particular towards sulphur donors, as observed by up to three platinum moieties attached to Cyt. The contrasting moderate reactivity of cisplatin (1) towards proteins points to different modes-of-action of the two Pt compounds, while biological data in a preliminary study suggests a comparable metabolic activity.^29^ Comparison of the respective Cyt and Ub adducts with compound 2 indicates differences in complex speciation, which presumably point toward a bidentate binding mode of the N-terminal Met residue in Ub and a mainly mono-dentate binding mode in Cyt. The time-resolved experimental setting revealed a loss of the chlorido ligand in the adduct of compound 2 with Ub after 6 to 12 h, resulting in the main adduct being of the composition [Ub + Pt(ala)]. With Cyt, no such loss of the chlorido ligand was observed, and [Cyt + Pt(ala)Cl] remained the main species after 24 h of incubation, while some of the ala ligand started to be cleaved, resulting in a low abundant adduct with the composition [Cyt + PtCl] suggesting a bi- or tridentate binding mode to the protein (Fig. S2).

**Fig. 2:**
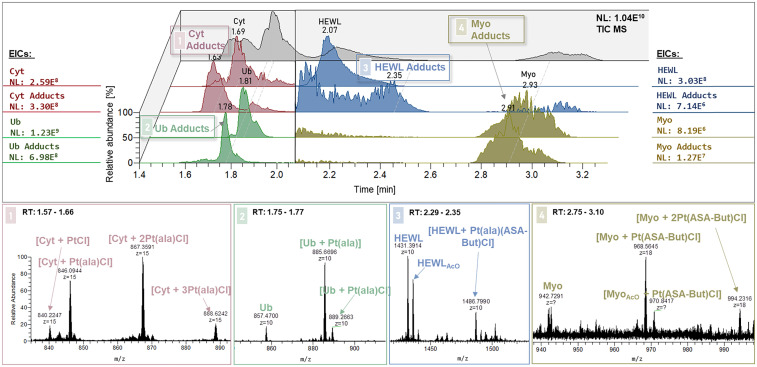
Top: Chromatograms of compound 2 with the protein mixture after 24 h incubation. TIC in black, extracted ion chromatograms (EICs) of proteins and protein adducts in red (Cyt), green (Ub), blue (HEWL), and ocre (Myo). Bottom: MS^1^ data of the protein adducts.

A more comprehensive examination of the speciation of compound 2 is presented in Fig. 2, which contains the observed adducts with all four proteins in the chromatograms and mass spectra. For a better overview of the elution behaviour of the metalated proteins, the TIC was split in the respective extracted ion chromatograms (EICs) of the proteins and protein adducts (Fig. 2, top). As mentioned before, Cyt formed multi-metalated adduct species with up to three Pt moieties attached to the protein. The adducts mostly retained the alanine (ala) and the chlorido ligands, while the ASA-But ligand was exchanged, which resulted in the adduct species [Cyt + *x*Pt(ala)Cl] (*x* = 1–3) and less abundant [Cyt + PtCl]. The doubly metalated species ([Cyt + 2Pt(ala)Cl]) showed the highest relative abundance, while one of the three S-binding sites in the Cyt sequence is less occupied, displayed by the lower signal for [Cyt + 3Pt(ala)Cl]. Ub formed a highly abundant singly metalated adduct, with the main adduct composition being [Ub + Pt(ala)] after the loss of the olefin and the chlorido ligand (Fig. 2, bottom). Upon Myo coordination the observed adducts showed the cleavage of the ala ligand, while ASA-But remained attached to the complex. In the HEWL adduct, complex 2 retained all of its original ligands. The difference in speciation in the formed adducts will be discussed in the section about binding site determination (*vide infra*).

#### HQ compounds (3 + 5)

The chelating HQ ligand was observed to hamper the reactivity to biomolecules, when compared to the NHC analogues. The Ru complex 3 showed only adduct formation with Ub and Myo, while the Ir compound was also observed to form a Cyt adduct (Fig. 1). Adducts of the composition [Prot + M(HQ)(Cym)/(Cp*)] (M = Ru, Ir) were detected for this compound class (Fig. S1 and Table S3).

#### NHC compounds (4 + 6)

The reactivity of the two NHC complexes was highly dependent on the nature of the biomolecule involved (*e.g.* number and accessibility of strongly donating amino acids, HSAB character, *etc.*). Compound 6 formed highly abundant mono- and bimetallic adduct species with Cyt and Ub with partial reduction of Ir^III^ to Ir^I^, while the Ru analogue 4 showed only moderate reactivity with less abundant singly metalated Ub and Cyt species. On the other hand, no adduct formation of compound 6 was observed with HEWL and Myo, whereas compound 4 showed higher reactivity towards Myo with doubly metalated adduct species. This partially agrees with a previous study, where, based on crystallization and MS data of HEWL with the NHC compounds (4, 6), the Ir-analogue was referred to as more inert.^9^ However, with Cyt and Ub the Ru compound was found to form fewer adducts than the Ir analogue, pointing out the strength of the competitive experimental approach to compare the reactivity in a more intricate chemical environment. Similar to compound 2, the speciation of 4 and 6 in the formed Cyt and Ub adducts was observed to be different. The loss of the NHC ligand happened upon Ub coordination ([Ub + Ru(cym)] and [Ub + Ir(Cp*)]), while no such ligand exchange was observed with Cyt (Fig. S1). Time-resolved analysis of the spectra showed that the loss of the NHC ligand in compound 4 happens much slower and to a lesser extent than in the Ir analogue (6) (Fig. S3). Cleavage of the NHC ligand in the Ub adducts was somewhat unexpected, given that previous studies reported the loss of the arene ligand rather than the NHC ligand upon interaction of compound 4 or analogues with HEWL.^34,36^ However, the NHC ligand in our experimental setup might exchange due to different chemical environments in Ub compared to HEWL.

**Fig. 3:**
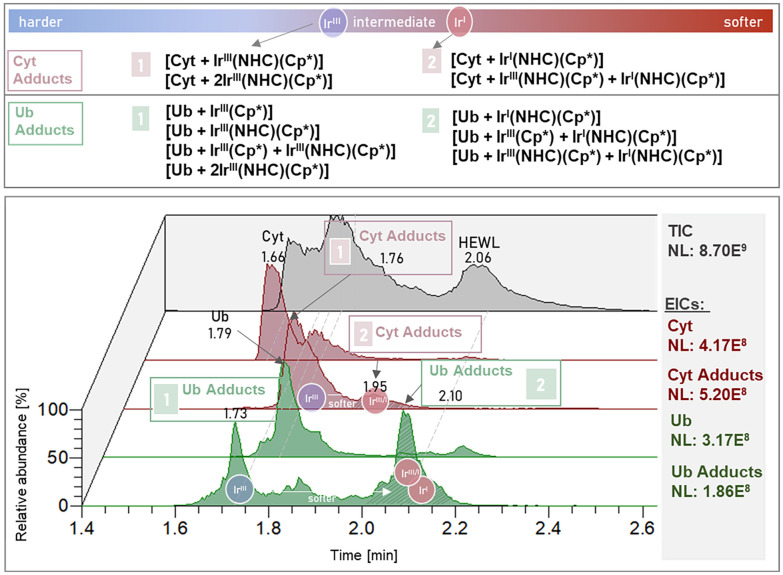
Bottom: Chromatogram of compound 6 incubated with the protein mixture for 24 h. TIC in black, EICs of proteins and protein adducts in red (Cyt) and green (Ub). Top: List of identified adducts at respective RTs.

Fig. 3 shows the TIC and EICs of proteins and protein adducts after 24 h incubation with compound 6, as well as a list of identified adducts. The influence of lipophilic ligands and the redox behaviour of Ir on the on-column retention did not allow for chromatographic separation between Cyt and Ub adducts in the incubation mixture with compound 6. The reduction of Ir^III^ to Ir^I^ was accompanied by a shift in retention time in both the Cyt and the Ub adducts (Fig. 3, Table S3 and Fig. S4). While free Cyt and Ub, as well as the mono-metalated adduct species ([prot + Ir(NHC)(Cp*)] (prot = Cyt, Ub)) were comparable in intensity, the bimetallic adduct species with Ub ([Ub + Ir(NHC)(Cp*) + Ir(NHC)_*x*_(Cp*)] (*x* = 0, 1)) were significantly more abundant than the Cyt analogous species ([Cyt + 2Ir(Cp*)(NHC)]). The loss of the NHC ligand upon interaction with Ub in the doubly metalated adduct was observed in only one of the two attached metal complexes, providing evidence for two different binding motifs within the Ub sequence (Fig. S4 and Table S3).

Reduction of Ir^III^ to Ir^I^ in compound 6 happened in the incubation with the protein mixture prior to adduct formation as seen in their relative areas in their TICs (see Fig. S5 and Table S4) with [Ir^III^(NHC)(Cp*)Cl]^+^ at a RT of 0.09 min and [Ir^I^(NHC)(Cp*) + H]^+^ at a RT of 1.35 min. Similarly, the protein adducts containing Ir^III^ and Ir^I^ species were well separated during the LC run (see Fig. 3). Reduction of compound 6 in the presence of peptides in aqueous solution was observed before.^37^ In contrast, the Ir(HQ) complex 5 retained its Ir^III^ oxidation state in all experiments and adducts.

The remarkable difference in affinity for particular proteins, especially between compound 2 and 6, can be rationalized with the HSAB concept. Considering the multiple sulphur-containing amino acids in the Cyt sequence, Pt^II^ readily formed multi-metalated Cyt adducts, while the harder character of Ir^III^ allowed for more coordination sites in Ub beyond the only sulphur containing Met residue. Ru^II^, as hardest metal centre, prefers the adduct formation with nitrogen containing amino acids.

### Binding site determination and mechanistic assumptions

To determine the metalation sites on the proteins, MS/MS experiments of the most interesting protein adducts were conducted (for fragment nomenclature see Fig. S6). All identified metalated fragments are listed in Table S5.

#### Platinum compounds (1 + 2)

For the cisplatin adduct with Cyt ([Cyt + Pt(NH_3_)_2_Cl]) the fragmentation pattern showed highly abundant [a/b65 + Pt − *x*H_2_O] (*x* = 0, 1) fragments and the non-metalated y39 as base peak (Fig. S7). This, together with a metalated y43 fragment indicate Met65 as binding site in Cyt. Upon fragmentation of the Ub adduct of cisplatin, all ligands were cleaved at NCE 20 and His68 was identified as binding site, shown by metalated C-terminal fragments (Fig. S8 and Table S5). Met1 is a known binding site for cisplatin in the Ub sequence,^32,35^ however, corresponding b fragments were not observed in this experimental setup.

**Fig. 4:**
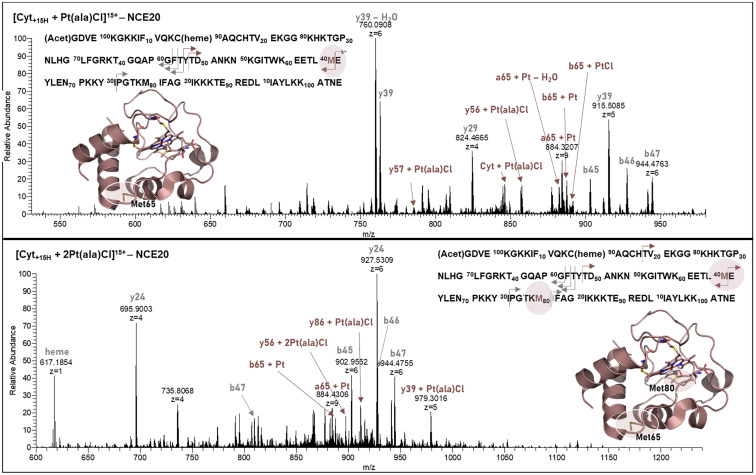
HCD fragmentation of Cyt adducts with compound 2 for [Cyt + Pt(ala)Cl] (top) and [Cyt + 2Pt(ala)Cl] (bottom) at a NCE (normalized collision energy) of 20. Metalated fragments are indicated in red, and non-metalated fragments are indicated in grey.

For compound 2, highly abundant platinated [a/b65 + Pt − *x*H_2_O] (*x* = 0, 1) fragments were identified in the fragmentation spectra of the mono- and bimetallic Cyt adducts, accompanied by a C-terminal y39 (breaks after Met65) fragment (Fig. 4 and Table S5). In the singly metalated adduct (Fig. 4, top), y39 as non-metalated species represents the base peak in the fragment spectrum, while in the bimetallic species (Fig. 4, bottom) the platinated [y39 + Pt(ala)Cl] fragment and non-metalated y24 (breaks after Met80) as the base peak were observed. This, together with the detection of [y56/57 + Pt(ala)Cl] from the singly metalated adduct and the bimetallic [y56 + 2Pt(ala)Cl] fragment in the doubly metalated adduct, indicate S_Met65_ as primary and S_Met80_ as secondary binding sites.

**Fig. 5:**
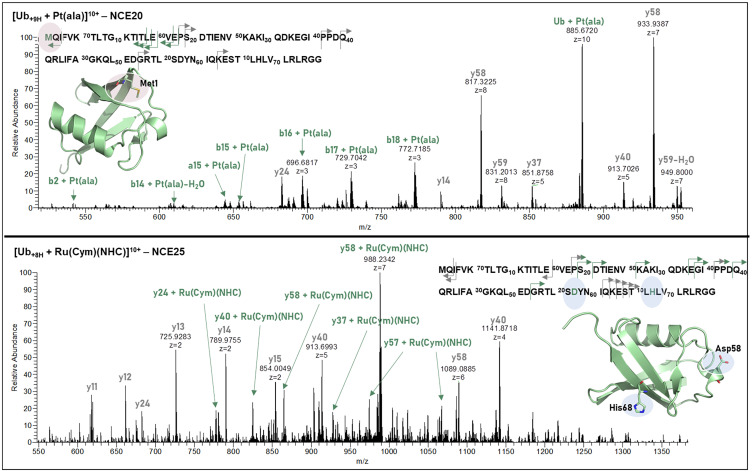
HCD fragmentation of Ub with compound 2 ([Ub + Pt(ala)] (top)) at a NCE of 20, and compound 4 ([Ub + Ru(Cym)(NHC)] (bottom)) at a NCE of 25 (fragmentation energies were optimized for the maximum amount of metalated peptide fragments and may differ for different systems). Metalated fragments are indicated in green, and non-metalated fragments are indicated in grey.

S_Met1_ was confirmed to be the primary binding site for compound 2 in Ub, indicated by the small metalated N-terminal b-fragment series [b/a*x* + Pt(ala)] (*x* = 14–18) and [b2 + Pt(ala)]^+^ (Fig. 5, top and Table S5).

**Scheme 2:**
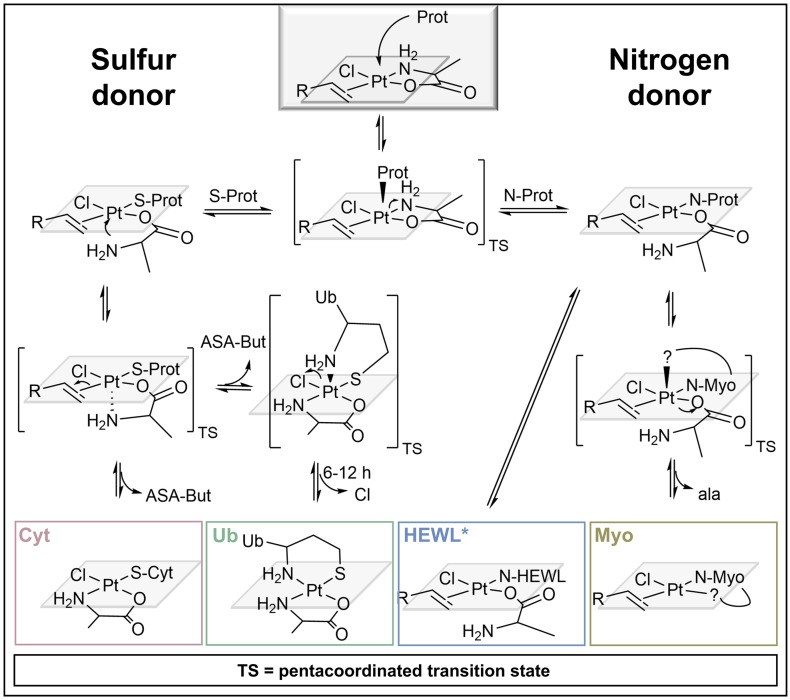
Proposed mechanisms for adduct formation of compound 2 with the four proteins.^38^ *Non-covalent adduct also possible.

The detailed investigation of binding sites of platinated protein adducts allowed for mechanistic assumptions, explaining the different speciation behaviour of compound 2 upon coordination to different proteins (Scheme 2). The attack of the protein donor atom led to a bipyramidal pentacoordinated transition state of the Pt complex. Due to the strong *trans*-effect of the olefin ligand, the Pt–N_ala_ coordination was weakened and cleaved predominantly, allowing the attacking nucleophilic residues to enter the square planar coordination sphere around the Pt^II^ centre. In case of sulphur donors (present in Cyt and Ub) the *trans*-effect of sulphur in turn led to the quick replacement of the olefin, through another pentacoordinated bipyramidal transition state and attack of N_ala_. The N-terminal Met in Ub subsequently replaced the chlorido ligand with N_Met1_ resulting in a bidentate binding motif.

If the incoming nucleophile was a nitrogen instead, the ASA-But ligand remained attached, because N-donors exhibit a much weaker *trans*-effect. The HEWL adduct [HEWL + Pt(ala)(ASA-But)Cl] retained all of the original ligands of compound 2, pointing to a monodentately coordinated ala-ligand or a non-covalent interaction between complex 2 and the protein. The cleavage of Pt–N_ala_ transformed ala into a good leaving ligand, as the coordination bond between oxygen as hard base and the soft Pt-centre is comparatively weak. Hence, we observed an exchange of the entire ala-ligand upon Myo coordination and recorded [Myo + *x*Pt(ASA-But)Cl] (*x* = 1, 2) adducts, in which the platinum centre presumably formed a bidentate coordination with a second suitable donor within the Myo sequence. These mechanistic assumptions represent our current understanding of these systems based on our LC-MS/MS experiments and previous studies by MS and NMR-spectroscopy.^38^

#### HQ compounds (3 + 5)

No metalated fragments could be identified for protein adducts of complexes 3 and 5. As only one chlorido leaving group is present in these compounds, only monodentate coordination bonds can be formed with biomolecules. These tend to cleave easily upon MS/MS, often with less fragmentation energy than is required for peptide backbone cleavage (Fig. S11).

#### NHC compounds (4 + 6)

A series of metalated C-terminal y fragments in the [Ub + Ru(Cym)(NHC)] fragment spectrum point to N_His68_ or O^δ1^_Asp58_ coordination of compound 4 (Fig. 5, bottom and Table S5), which is in good agreement with the HSAB concept. Also, His has been identified as preferred binding site in HEWL for compound 4 in a previous study.^9^ For the Cyt adduct with compound 4 some metalated N-terminal fragments point to His26 or His33 as binding site (Fig. S9 and Table S5). For the Ir analogue (6) no metalated fragments were observed in the mono adduct, as the complex cleaved off the proteins at relatively low fragmentation energies (Fig. S11). However, as we observed the above mentioned doubly metalated Ub adduct, which is the only observed bimetallic Ub species within all investigated compounds, we assume coordination of both S_Met1_ and N_His68_, agreeing with the intermediate HSAB character of Ir. Some low-abundant metalated N-terminal fragments with the composition [*bx* + Ir(Cp*)] (*b* = 16–18) could be identified for [Ub + Ir(NHC)(Cp*) + Ir(Cp*)], indicating a bidentate coordination of S_Met1_ (Fig. S10 and Table S5).

The donor atom involved in the interaction significantly influenced the biospeciation of metal complexes, as was seen most pronounced for compound 2. This behaviour highlights the potential to exploit these speciation differences for bioaccessibility and target specificity. On the other hand, the reactivity and binding modes of the compounds with certain biomolecules seems to be tuneable through the HSAB character of the metal ion and the chemical properties of the surrounding ligands, counteracting the prejudice of their promiscuity.

### Nucleophile exposure

**Fig. 6:**
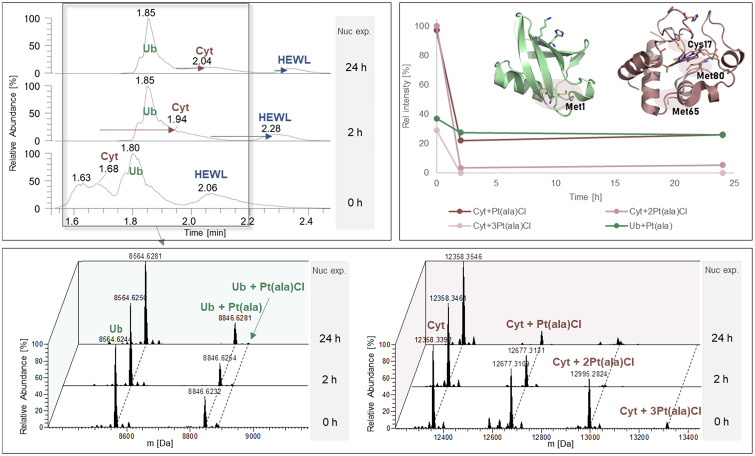
Nucleophile exposure of protein adducts of compound 2. Top, left: TIC after 0, 2, and 24 h nucleophile exposure (RT window for calculation of deconvoluted spectra highlighted in grey). Top, right: time-resolved plot of protein adduct cleavage relative to the free protein. Bottom: Deconvoluted spectra of Ub and Cyt adducts after 0, 2, and 24 h nucleophile exposure.

After 24 h incubation of the protein mixture with compounds 1–6, any excess of unreacted complex was removed by a size cut-off filter and an eight-fold excess of an equimolar GSH and Met mixture was added. The incubation was then continued at 20 °C for another 24 h. Time-resolved LC-MS measurements were conducted to track the behaviour of the metalated proteins when exposed to strong nucleophiles. The decline of protein adducts was observed for all complexes to a similar extent, while ternary adduct species of the type nucleophile-metallodrug-protein were not observed in any of the experiments. Comparing the chromatograms of 0, 2, and 24 h nucleophile exposure, we observed a shift in Cyt and HEWL elution time accompanied by a significant decrease in intensity (Fig. 6, top left), which was detected in all spectra (compound 1–6) after adding the nucleophiles. As the charge state distribution showed no shift towards higher *m*/*z* values, reduction of disulphide bonds was excluded as reason for the shift in retention time. Hence, as the most likely explanation, the 3D structure of the protein was altered due to pH changes upon nucleophile addition, altering the elution behaviour of the proteins.

Fig. 6 shows the TICs of compound 2 after 0, 2, and 24 h nucleophile exposure, together with the deconvoluted spectra of Cyt and Ub calculated for the indicated retention time (grey square, Fig. 6). The detoxification reaction was traced in a time-resolved plot as shown in Fig. 6, top right. We observed nearly quantitative conversion of the bi- and trimetallic Cyt adduct species of compound 2 within 2 h at 20 °C, indicating a rapid interaction with the nucleophiles. The Cyt and Ub mono-adducts were more persistent and the nucleophile interaction was found to be in equilibrium after 2 h nucleophile exposure. Despite the absence of detected nucleophile-metallodrug adducts, the decrease in signal intensity of the Pt-protein adducts relative to the free proteins clearly points to rapid interactions with the added nucleophiles (Table S6). The chromatographic separation of the excess of nucleophiles from the proteins and protein adducts excludes ion suppression as interfering experimental parameter.

**Fig. 7:**
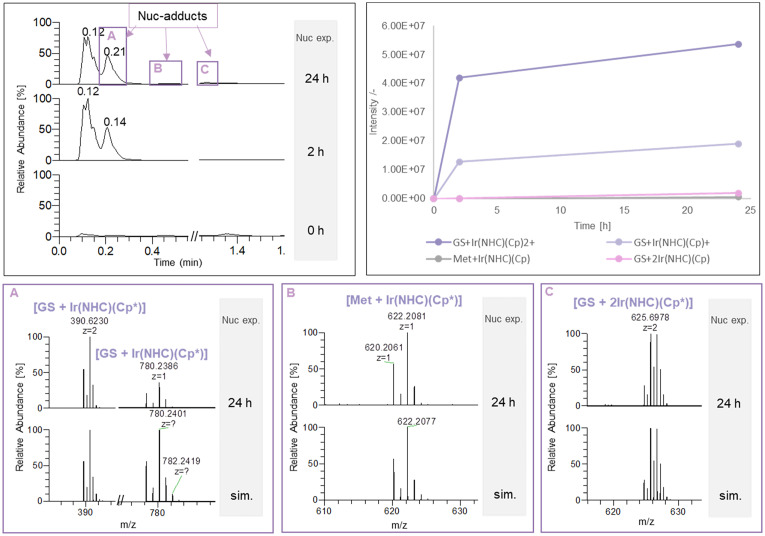
Nucleophile adducts of compound 6. Top, left: TIC after 0, 2, and 24 h nucleophile exposure. Top, right: time-resolved intensities of nucleophile-metallodrug adducts with compound 6. Bottom: Mass spectra of the 24 h nucleophile exposure measurement and simulated isotope patterns of [GS + Ir(NHC)(Cp*)] (A), [Met + Ir(NHC)(Cp*)] (B), and [GS + 2Ir(NHC)(Cp*)] (C).

Nucleophile-metallodrug adducts were mainly observed with the Ir compounds (5, 6), while only low-abundance adduct species could be identified for Ru compounds (3, 4) (Table S7 and Fig. S12–S14). For both Pt compounds (1, 2), nucleophile-metallodrug adducts were not detected. Especially compound 6 was observed to form multiple GSH and Met adducts with distinct retention times in the chromatogram (Fig. 7). The interaction with stronger nucleophiles is governed by ligand exchange rates and in-solution equilibria and thus, the binding motif and ligand shielding of the initial protein adduct matters. The protein adducts of compound 6 with Cyt and Ub were mainly detected as [prot + Ir(NHC)(Cp*)], providing a vacancy as both Cl^−^ ligands were replaced, facilitating the nucleophilic attack. For compound 5 instead, the bidentate 8-oxyquinolinato ligand in [prot + Ir(HQ)(Cp*)] is more strongly bound due to the chelate effect and limits access to the metal centre.

The most abundant adduct peak with small nucleophiles was found to be [GS + Ir(NHC)(Cp*)] (Fig. 7A). An unexpected bimetallic GSH adduct was observed with compound 6 ([GS + 2Ir(NHC)(Cp*)]; Fig. 7C and Table S7), indicating a second binding site within the GSH sequence or the formation of a sulphur bridging bis-coordination. [Met + Ir(NHC)(Cp*)] (Fig. 7B) was the only identified Met adduct within all complexes. The fact that the observed nucleophile adducts elute with distinct retention times suggests that these adduct species form in the reaction solution.

This model study shows rapid interaction of metalated protein adducts with strong nucleophiles independent of the formation of stable nucleophile adducts. Despite the high excess of nucleophiles some protein adducts are persistent against nucleophilic attacks. The fact that compound 6 formed stable nucleophile adduct species with up to two complexes attached to GSH, while the other complexes showed little to no adduct formation indicates a very distinct kinetic behaviour of Ir, which is worth of further studying in detail.

### Limitations of the study

This study introduced a time-dependent and competitive top-down LC-MS/MS approach to probe the interaction of reactive metallodrugs with a mixture of intact proteins to reveal the nature, selectivity, and location of metallodrug-protein adducts. It must be noted that this model system was designed to facilitate the MS-based analysis of metallodrug-protein adducts and does not entirely mimic biological conditions, which has several ramifications. First, the selected proteins should have adequate concentrations and a molecular mass of <50 000 Da to qualify for online top-down fragmentation. Second, the proteins in this study were deliberately chosen to not represent potential targets so that adduct formation can be largely interpreted as off-target binding. Yet, the presence of numerous surface-accessible nucleophilic amino acids in varying micro-environments enables testing a biologically meaningful competitive reactivity. It is known that reactive metallodrugs tend to form adducts with numerous nucleophiles when exposed in isolation,^39^ but show binding selectivity when employing competitive assays.^40^ The competitive character of the assay is crucial to investigate binding selectivity, and the impact of different reaction equivalents and ratios may be further investigated. Follow-up studies will also increase the complexity of the protein mixture, *i.e.* including biologically relevant targets, as well as aim for physiologically more realistic conditions while maintaining analytical depth.

Finally, reactions were carried out in aqueous solution in the present model system, similarly to earlier studies.^40^ This is partly an instrumental limitation, as non-volatile buffers are generally avoided in MS-based assays due to their disruption of ion formation, transmission, and detection and potential damage to the instrument. These include chlorides and phosphates. More importantly, however, buffers have a dual impact on the reactivity of metallodrugs that rely on ligand exchange for their activation. After hydrolysis of the metal–chloride bond and formation of the aqua complex, slightly acidic conditions increase ligand exchange rates because the aqua-ligand is slightly acidic itself, while basic conditions favour deprotonation of the aqua-ligand to form a kinetically unreactive hydroxido-complex. The p*K*_a_ of the aqua-complex can be tuned by the metal and the inert (chelating) ligand.^41^ Importantly, a comparison of aqueous solution reactivity *versus* different buffer reactivity has revealed mainly similar adduct types for cisplatin, but aqueous solution was found to accelerate reaction rates compared to buffered systems.^42^ It was also shown in the same study that buffer components would directly interact with the metallodrug and form phosphate- and ammine-adducts.

For the Ru compounds 3 and 4, experiments were repeated under buffered conditions (20 mM tetraethylammonium bicarbonate, pH 8) and confirmed the above considerations. For complex 3, the same adducts were found as in aqueous solution, only to a larger extent (see Fig. S15). The reaction with the nucleophiles also led to the same adduct in both conditions (see Fig. S16). Complex 4 on the other hand was completely inactive in buffered conditions due to deprotonation of the aqua-complex to the unreactive hydroxide-complex (see Fig. S15).

## Conclusion

In this study, we investigated the metalation dynamics of metal compounds with Pt, Ru, and Ir centres with 4 different model proteins in a competitive, time-resolved setting and subsequent exposure to an excess of small nucleophiles. This approach aimed to directly compare and evaluate specific reactivities of metallodrugs in a competitive, rather than isolated setting, using LC-MS and top-down LC-MS/MS to identify binding sites. The lack of specificity of metallodrugs is among the main bottlenecks for their application in a pharmaceutical context. Understanding and tailoring the extent of these molecular interactions to further optimize their pharmacological behaviour and thus, fully exploit their chemical space, is a major goal in advanced inorganic medicinal chemistry. The study design allowed for investigating coordination and speciation behaviour of metal-based drugs in a competitive model setting, while simultaneously gaining information about binding sites and motifs, as well as detoxification dynamics. The obtained results indicate differences in complex speciation depending on the chemical environment of the biomolecule. This information combined with the influence of the coordinated ligands and the HSAB character of the metal ions, can be exploited for the design of advanced metallodrugs.

Detailed top-down MS/MS analysis revealed that the Zeise's salt derivative, Pt(ala)(ASA-But)Cl, showed high affinity towards sulphur donors and especially Met residues, leading to up to triply metalated Cyt adducts and a highly abundant mono-metalated bidentate Ub adduct. The reactivity of the organometallic Pt compound 2 was found to be significantly increased compared to cisplatin. The strong *trans*-effect of the olefin ligand causes high reactivity, which is modulated by the presence of the bidentate l-alanine ligand, leading to different speciation and adduct formation depending on the nature of the donor atom in the protein. In line with the HSAB concept, Ru compounds preferred interaction with His residues in Ub and Myo. The ability of Ir to form doubly metalated Ub adducts indicated an interaction with both sulphur and nitrogen donor atoms, accompanied by partial reduction of one of the two attached Ir ions. The HQ ligand in Ir and Ru complexes 3 and 5 hampered their reactivity towards proteins and nucleophiles. In general, our findings demonstrate the influence of the ancillary ligands on the speciation of metal complexes, which in turn governs their ability to interact specifically with one or more target molecules. The slightly redox-active Ir compounds were found to form protein adducts that subsequently were cleaved by nucleophiles and formed stable nucleophile-metallodrug adducts, *e.g.* a doubly metalated GSH adduct. This finding can be rationalized by the presence of both a thiol and a primary amine acting as potential donors for Ir in the [GS + 2Ir(NHC)(Cp*)] adduct, or by bridging coordination of one donor atom. The initial binding motif of the formed adducts and the accessibility of the metal centre influence their stability and the propensity to be attacked by nucleophiles.

This methodological approach can be applied to a variety of different metal complexes to predict reactivities of metallodrugs with a model system such as above or more tailored towards a specific target. The systematic tracking and evaluation of reactivities on the molecular level represents an important step towards rational drug design approaches.

## Author contributions

Kira Küssner: writing – original draft, conceptualization, investigation, formal analysis, visualization. Michael Wolf: investigation, methodology, writing – review & editing. Andrea Cucchiaro: resources. Christian Hartinger: resources, writing – review & editing. Samuel Meier-Menches: conceptualization, methodology, writing – review & editing, supervision. Monika Cziferszky: writing – review & editing, conceptualization, supervision, funding acquisition.

## Conflicts of interest

The authors declare that they have no known competing financial interests or personal relationships that could have appeared to influence the work reported in this paper.

## Supplementary Material

QI-013-D5QI02457A-s001

## Data Availability

The data supporting this article have been included as part of the supplementary information (SI). Supplementary information: Tables S1–S7, time-resolved LC-MS spectra, deconvoluted spectra, and MS/MS spectra. See DOI: https://doi.org/10.1039/d5qi02457a. Additional data will be made available on request.
