# Supplementary material for: Competitive LC-MS/MS assay to investigate protein metalation dynamics
Source: Inorg Chem Front. 2026 Feb 19;13(8):3574–86. doi: 10.1039/d5qi02457a (PMC12935058; doi:10.1039/d5qi02457a)
Supplement: QI-013-D5QI02457A-s001 [file QI-013-D5QI02457A-s001.pdf]

Supplementary Information (SI) for Inorganic Chemistry Frontiers.
This journal is © the Partner Organisations 2026

Supporting information

Competitive LC-MS/MS Assay to Investigate Protein Metalation Dynamics

Kira Küssner,[1] Michael Wolf,[2,3] Andrea Cucchiaro,[1] Christian G. Hartinger,[4] Samuel Meier-Menches,[2,5,6*] Monika Cziferszky[1*]

[1] Institute for Pharmacy, Pharmaceutical Chemistry, Department of Chemistry and Pharmacy, Center for Molecular Bioscience (CMBI), University of Innsbruck, Innrain 80/82, A-6020 Innsbruck, Austria.
[2] Institute of Chemistry, Analytical Chemistry, University of Vienna, Währinger Straße 38, A-1090 Vienna, Austria.
[3] Doctoral School in Chemistry, University of Vienna, Waehringer Str. 38, A-1090 Vienna, Austria.
[4] Faculty of Science, Chemical Science, University of Auckland, Science Centre 302 – Bldg 302, 23 Symonds St, Auckland Central, 1010 Auckland, New Zealand.
[5] Institute of Inorganic Chemistry, Faculty of Chemistry, University of Vienna, Waehringer Str. 42, A-1090 Vienna, Austria.
[6] Joint Metabolome Facility, Medical University of Vienna and University of Vienna, Waehringer Str. 38, A-1090 Vienna, Austria.

Table S1. Formulae and isotopologues throughout the analysis.

Table S2. Protein Sequences with potential binding sites highlighted.

1) LC-MS data for the reactions of the protein mix with compounds 1 – 6:

Figure S1. TICs after 24 h incubation of the protein mix and the protein-compound mixtures.

Table S3. Identified protein adducts: m/z values, errors, RT and signal intensity.

2) Time-resolved adduct formation

Figure S2. Time resolved mass spectra of compound **2** with Cyt and Ub.

Figure S3. Time-resolved mass spectra of Ub with compound **4** and compound **6**.

3) Complex and adduct speciation

Figure S4. Deconvoluted mass spectra of compound **6** with Cyt and Ub.

Figure S5. Speciation of compound **6** after incubation with the protein mixture.

Table S4. Identified free complex species of compound **6** with sum formula, m/z, error and RT

4) LC-MS/MS-fragmentation of selected precursor ions:

Figure S6. MS/MS fragment nomenclature of proteins.

Table S5. Identified MS/MS-fragments: m/z values, errors, RT and signal intensity.

Figure S7. HCD fragmentation spectrum of [Cyt + Pt(NH3)2Cl] at NCE 20 with corresponding protein sequence and 3D structure of the protein.

Figure S8. HCD fragmentation spectrum of [Ub + $Pt(NH_3)_2Cl$] at NCE 20 with corresponding protein sequence and 3D structure.

Figure S9. HCD fragmentation spectrum of [Cyt + Ru(NHC)(Cym)] at NCE 20 with corresponding protein sequence and 3D structure.

Figure S10. HCD fragmentation spectrum of [Ub + Ir(NHC)(Cp*) + Ir(Cp*)] at NCE 20 and 25 with corresponding protein sequence and 3D structure.

Figure S11. HCD fragmentation spectra of protein adducts without metalated fragments (compound **3**, **5**, and **6**) at NCE 20 and 25 with corresponding protein sequence and 3D structure.

5) Nucleophile exchange of compound 2 and 6

Table S6. Adduct reduction of compound **2** and **6** with Cyt and Ub after 0, 2, and 24 h nucleophile exposure.

6) Nucleophile adducts of compounds 1 – 6

Table S7. Identified nucleophile adducts with the compounds: m/z values, errors, RT and signal intensity.

Figure S12. Nucleophile adduct with compound **3**.

Figure S13. Nucleophile adduct with compound **4**.

Figure S14. Nucleophile adducts with compound **5**.

6) Experiments in buffered solution

Figure S15. TICs after 24 h incubation of the protein mixtures with **3** and **4** in aqueous solution and 20 mM tetraethylammonium bicarbonate.

Figure S16. Comparison of the nucleophile adduct with compound **3** formed in aqueous solution and 20 mM tetraethylammonium bicarbonate.

Table S1. Formulae and isotopologues throughout the analysis.

Protein	Formulae & isotopologues	complex	Formulae & isotopologues
**Cyt***	$C_{560} H_{873} N_{148} O_{156} S_4 {}^{56}Fe_1$	**1** – $Pt(NH_3)_2Cl_2$	${}^{195}Pt\ N_2\ H_6\ {}^{35}Cl_2$
**Ub**	$C_{378} H_{629} N_{105} O_{118} S_1$	**2** – Pt(ala)(ASA-But)Cl	${}^{195}Pt\ C_{16} H_{20}\ N\ O_6\ {}^{35}Cl$
**HEWL**	$C_{613} H_{951} N_{193} O_{185} S_{10}$	**3** – Ru(HQ)(Cym)Cl	${}^{102}Ru\ C_{19} H_{20}\ N\ O$
**Myo**	$C_{769} H_{1212} N_{210} O_{218} S_2$	**4** – $Ru(NHC)(Cym)Cl_2$	${}^{102}Ru\ C_{19} H_{24} N_2\ {}^{35}Cl_2$
**GSH**	$C_{10} H_{17} N_3 O_6\ S$	**5** – Ir(HQ)(Cp*)Cl	${}^{193}Ir\ C_{19} H_{21}\ N\ O\ {}^{35}Cl$
**Met**	$C_5 H_{11}\ N\ O_2\ S$	**6** – $Ir(NHC)(Cp^*)Cl_2$	${}^{193}Ir\ C_{19} H_{25} N_2\ {}^{35}Cl_2$

* (+ Ac + heme)

Table S2. Protein Sequences with potential binding sites highlighted.

Protein	Sequence
**Cyt***	(Acet)GDVEKGKKIF$_{10}$ VQK**C$_{14}$**(heme)AQ**C$_{17}$**(heme)HTV$_{20}$ EKGGK**H$_{26}$**KTGP$_{30}$ NL**H$_{33}$**GLFGRKT$_{40}$ GQAPGFTYTD$_{50}$ ANKNKGITWK$_{60}$ EETL**M$_{65}$**EYLEN$_{70}$ PKKYIPGTK**M$_{80}$** IFAGIKKKTE$_{90}$ REDLIAYLKK$_{100}$ ATNE
**Ub**	**M$_1$**QIFVKTLTG$_{10}$ KTITLEVEPS$_{20}$ **D$_{21}$**TIENVKAKI$_{30}$ QDKEGIPP**D$_{39}$**Q$_{40}$ QRLIFAGKQL$_{50}$ E**D$_{52}$**GRTLS**D$_{58}$**YN$_{60}$ IQKESTL**H$_{68}$**LV$_{70}$ LRLRGG
**HEWL**	KVFGRCELAA$_{10}$ AMKR**H$_{15}$**GLDNY$_{20}$ RGYSLGNWVC$_{30}$ AA**K$_{33}$**FESNFNT$_{40}$ QATNRNTDGS$_{50}$ TDYGILQINS$_{60}$ RWWCNDGRTP$_{70}$ GSRNLCNIPC$_{80}$ SALLSSDITA$_{90}$ SVNCAKKIVS$_{100}$ **D$_{101}$**GNGMNAWVA$_{110}$ WRNRCKGTDV$_{120}$ QAWIRGCRL
**Myo**	GLSDGEWQQV$_{10}$ LNVWGKVEAD$_{20}$ IAG**H$_{24}$**GQEVLI$_{30}$ RLFTG**H$_{36}$**PETL$_{40}$ EKFDKFK**H$_{48}$**LK$_{50}$ TEAEMKASED$_{60}$ LKK**H$_{64}$**GTVVLT$_{70}$ ALGGILKKKG$_{80}$ **H$_{81}$H$_{82}$**EAELKPLA$_{90}$ QS**H$_{93}$** ATK**H$_{97}$**KIP$_{100}$ IKYLEFISDA$_{110}$ II**H$_{113}$**VL**H$_{116}$**SK**H$_{119}$**P$_{120}$ GDFGADAQGA$_{130}$ MTKALELFRN$_{140}$ DIAAKYKELG$_{150}$ FQG

* (+ Ac + heme)

1) LC-MS data for the reactions of the protein mix with compounds 1 – 6:

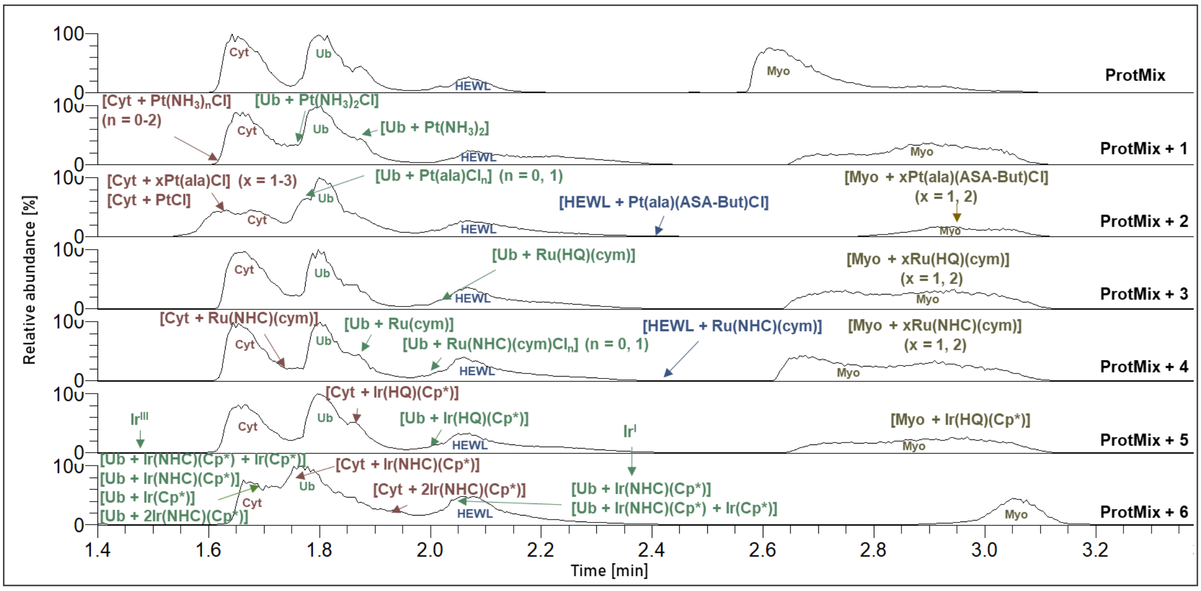

Figure S1. TICs after 24 h incubation of the protein mix and the protein-compound mixtures (**1** – $[Pt^{II}(NH_3)_2Cl_2]$; **2** – $[Pt^{II}(ala)(ASA\text{-}But)Cl]$; **3** – $[Ru^{II}(HQ)(cym)Cl]$; **4** – $[Ru^{II}(NHC)(cym)Cl_2]$; **5** – $[Ir^{III}(HQ)(Cp^*)Cl]$; **6** – $[Ir^{III}(NHC)(Cp^*)Cl_2]$). Identified adducts are indicated at their respective retention times.

Table S3. Identified protein adducts: m/z values, mass errors, RT, and signal intensity (Cyt – red, Ub – green, HEWL – blue, Myo – ocre; adducts considered for MS/MS inclusion list highlighted in grey). Both m/z_{exp} and m/z_{theo} refer to the basepeak in the respective cluster of isotopologues.

Complex	Adduct	m/z_{exp} /-	m/z_{theo} /-	Error /ppm	RT /min	Intensity /-
**1** $Pt(NH_3)_2Cl_2$	**$[Cyt^*_{+15H}]^{15+}$**	**824.8951**	**824.8963**	**-1.45**	**1.66**	**9.28E7**
	$[Cyt^*_{+14H} + Pt^{II}Cl^{(1+)}]^{15+}$	840.0932	840.0914	2.14	1.62	1.88E6
	$[Cyt^*_{+14H} + Pt^{II}(NH_3)Cl^{(1+)}]^{15+}$	841.3596	841.3599	-0.36	1.63	2.44E6
	$[Cyt^*_{+14H} + Pt^{II}(NH_3)_2Cl^{(1+)}]^{15+}$	842.4291	842.4282	1.07	1.63	3.49E6
**2** Pt(ala)(ASA-But)Cl	**$[Cyt^*_{+15H}]^{15+}$**	**824.8964**	**824.8963**	**0.12**	**1.68**	**2.26E7**
	$[Cyt^*_{+14H} + Pt^{II}Cl^{(1+)}]^{15+}$	840.2245	840.2246	-0.12	1.58	4.20E6
	$[Cyt^*_{+15H} + Pt^{II}(ala)Cl^{(0)}]^{15+}$	846.0944	846.0943	0.12	1.64	2.20E7
	$[Cyt^*_{+15H} + 2Pt^{II}(ala)Cl^{(0)}]^{15+}$	867.3591	867.3588	0.35	1.61	2.27E7
	$[Cyt^*_{+15H} + 3Pt^{II}(ala)Cl^{(0)}]^{15+}$	888.6244	888.6242	0.23	1.60	6.61E6
**4** $Ru(NHC)(cym)Cl_2$	**$[Cyt^*_{+15H}]^{15+}$**	**824.8956**	**824.8963**	**-0.85**	**1.64**	**1.22E8**
	$[Cyt^*_{+13H} + Ru^{II}(NHC)(cym)^{(2+)}]^{15+}$	850.2346	850.2352	-0.71	1.75	9.33E6
**5** Ir(HQ)(Cp*)Cl	**$[Cyt^*_{+15H}]^{15+}$**	**824.8962**	**824.8963**	**-0.12**	**1.65**	**1.09E8**
	$[Cyt^*_{+13H} + Ir^{III}(HQ)(Cp^*)^{(1+)}]^{14+}$	917.3965	917.3967	-1.22	1.86	7.75E6
**6** $Ir(NHC)(Cp^*)Cl_2$	**$[Cyt^*_{+15H}]^{15+}$**	**824.8958**	**824.8963**	**-0.61**	**1.66**	**6.05E7**
	$[Cyt^*_{+12H} + Ir^{III}(NHC)(Cp^*)^{(2+)}]^{14+}$	917.4700	917.4704	-0.44	1.76	6.75E7
	$[Cyt^*{+13H} + Ir^{I}(NHC)(Cp^*)^{(0)}$ $^{+}Ir^{III}(NHC)(Cp^*)^{(2+)}]^{15+}$	887.9168	887.9174	-0.68	1.98	2.23E6
**1** $Pt(NH_3)_2Cl_2$	**$[Ub_{+10H}]^{10+}$**	**857.4679**	**857.4703**	**-2.80**	**1.80**	**2.74E8**
	$[Ub_{+7H} + Pt^{II}(NH_3)_2^{(2+)}]^{9+}$	977.8529	977.8551	-2.25	1.86	2.84E6
	$[Ub_{+8H} + Pt^{II}(NH_3)_2Cl^{(1+)}]^{9+}$	981.9622	981.9638	-1.63	1.77	8.19E6
**2** Pt(ala)(ASA-But)Cl	**$[Ub_{+10H}]^{10+}$**	**857.4694**	**857.4703**	**-1.05**	**1.80**	**2.09E8**
	$[Ub_{+9H} + Pt^{II}(ala)^{(1+)}]^{10+}$	885.6696	885.6699	-0.34	1.78	1.22E8
	$[Ub_{+10H} + Pt^{II}(ala)Cl^{(0)}]^{10+}$	889.2664	889.2679	-1.69	1.75	8.67E6
**3** Ru(HQ)(cym)Cl	**$[Ub_{+10H}]^{10+}$**	**857.4689**	**857.4703**	**-1.63**	**1.80**	**2.74E8**
	$[Ub_{+9H} + Ru^{II}(HQ)(cym)^{(1+)}]^{10+}$	895.3746	895.3756	-1.12	1.98	4.22E6

**4**<br>Ru(NHC)(cym)Cl$_2$	**[Ub$_{+10H}$]$^{10+}$**	**857.4690**	**857.4703**	**-1.52**	**1.80**	**2.38E8**
	[Ub$_{+8H}$ + Ru^{II}(cym)$^{(2+)}$]$^{10+}$	880.8690	880.8703	-1.48	1.83	4.18E6
	[Ub$_{+8H}$ + Ru^{II}(NHC)(cym)$^{(2+)}$]$^{10+}$	895.4777	895.4783	-0.67	1.90	7.83E6
	[Ub$_{+9H}$ + Ru^{II}(NHC)(cym)Cl$^{(1+)}$]$^{10+}$	899.0768	899.0763	0.56	1.98	2.03E6
**5**<br>Ir(HQ)(Cp*)Cl	**[Ub$_{+10H}$]$^{10+}$**	**857.4696**	**857.4703**	**-0.82**	**1.80**	**2.67E8**
	[Ub$_{+9H}$ + Ir^{III}(HQ)(Cp*)$^{(1+)}$]$^{10+}$	904.4817	904.4819	-0.22	1.99	1.81E6
**6**<br>Ir(NHC)(Cp*)Cl$_2$	**[Ub$_{+10H}$]$^{10+}$**	**857.4693**	**857.4703**	**-1.17**	**1.79**	**5.55E7**
	[Ub$_{+8H}$ + Ir^{III}(Cp*)$^{(2+)}$]$^{10+}$	889.9761	889.9766	-0.56	1.71	1.49E7
	[Ub$_{+8H}$ + Ir^{III}(NHC)(Cp*)$^{(2+)}$]$^{10+}$	904.5847	904.5850	-0.33	1.73	2.06E7
	[Ub$_{+6H}$ + Ir^{III}(NHC)(Cp*)$^{(2+)}$ + Ir^{III}(Cp*)$^{(2+)}$]$^{10+}$	937.1918	937.1917	0.11	1.71	1.51E7
	[Ub$_{+5H}$ + 2Ir^{III}(NHC)(Cp*)$^{(2+)}$]$^{9+}$	1057.4439	1057.4438	0.09	1.66	3.47E7
	[Ub$_{+10H}$ + Ir^{I}(NHC)(Cp*)$^{(0)}$]$^{10+}$	904.7860	904.7867	-0.77	2.09	2.15E7
	[Ub$_{+8H}$ + Ir^{I}(NHC)(Cp*)$^{(0)}$ + Ir^{III}(Cp*)$^{(2+)}$]$^{10+}$	937.3938	937.3933	0.53	2.00	2.61E6
	[Ub$_{+7H}$ + Ir^{I}(NHC)(Cp*)$^{(0)}$ + Ir^{III}(NHC)(Cp*)$^{(2+)}$]$^{9+}$	1057.6671	1057.6675	-0.38	2.10	2.53E5
**2**<br>Pt(ala)(ASA-But)Cl	**[HEWL$_{+10H}$]$^{10+}$**	**1431.3896**	**1431.3904**	**-0.56**	**2.06**	**4.45E7**
	[HEWL$_{AcO\ +10H}$]$^{10+}$	1435.6892	1435.6926	-2.37	2.06	1.00E7
	[HEWL$_{+10H}$ + Pt^{II}(ala)(ASA-But)Cl$^{(0)}$]$^{10+}$	1486.6990	1486.6970	1.35	2.35	1.06E6
**4**<br>Ru(NHC)(cym)Cl$_2$	**[HEWL$_{+10H}$]$^{10+}$**	**1431.3883**	**1431.3901**	**-1.28**	**2.06**	**1.06E8**
	[HEWL$_{+8H}$ + Ru^{II}(NHC)(cym)$^{(2+)}$]$^{10+}$	1469.4006	1469.3991	1.02	2.39	5.61E5
**2**<br>Pt(ala)(ASA-But)Cl	**[Myo$_{+18H}$]$^{18+}$**	**942.7294**	**942.7291**	**0.32**	**2.94**	**2.08E5**
	[Myo$_{+17H}$ + Pt^{II}(ASA-But)Cl$^{(1+)}$]$^{18+}$	968.5075	968.5076	-0.10	2.92	6.66E5
	[Myo$_{AcO\ +17H}$ + Pt^{II}(ASA-But)Cl$^{(1+)}$]$^{18+}$	970.8416	970.8415	0.10	2.94	1.54E5
	[Myo$_{+16H}$ + 2Pt^{II}(ASA-But)Cl$^{(1+)}$]$^{18+}$	994.2865	994.2875	-1.01	2.95	1.84E5
**3**<br>Ru(HQ)(cym)Cl	**[Myo$_{+18H}$]$^{18+}$**	**942.7286**	**942.7292**	**-0.64**	**2.75**	**8.57E6**
	[Myo$_{+17H}$ + Ru^{II}(HQ)(cym)$^{(1+)}$]$^{18+}$	963.7876	963.7877	-0.10	2.77	4.34E6
	[Myo$_{+16H}$ + 2Ru^{II}(HQ)(cym)$^{(1+)}$]$^{18+}$	984.7903	984.7901	0.20	2.89	3.44E6
**4**<br>Ru(NHC)(cym)Cl$_2$	**[Myo$_{+18H}$]$^{18+}$**	**942.7304**	**942.7292**	**1.27**	**2.66**	**8.48E6**
	[Myo$_{+16H}$ + Ru^{II}(NHC)(cym)$^{(2+)}$]$^{18+}$	963.8461	963.8445	1.66	2.67	1.39E7
	[Myo$_{+14H}$ + 2Ru^{II}(NHC)(cym)$^{(2+)}$]$^{18+}$	984.9064	984.9048	1.62	2.68	3.41E6
**5**<br>Ir(HQ)(Cp*)Cl	**[Myo$_{+18H}$]$^{18+}$**	**942.7305**	**942.7291**	**1.49**	**2.71**	**8.78E6**
	[Myo$_{+17H}$ + Ir^{III}(HQ)(Cp*)$^{(1+)}$]$^{18+}$	986.8486	968.8461	2.58	2.94	8.96E5

* (+ Ac + heme)

2) Time-resolved adduct formation

*Compound **2**.*

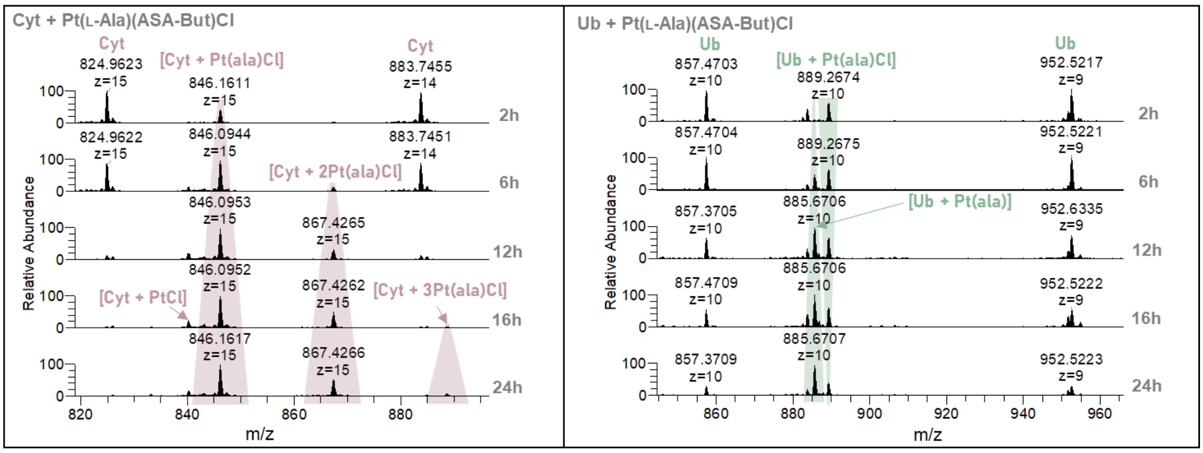

Figure S2. Time-resolved mass spectra of compound **2** with Cyt (left) and Ub (right).

*NHC-compounds (**4** + **6**):*

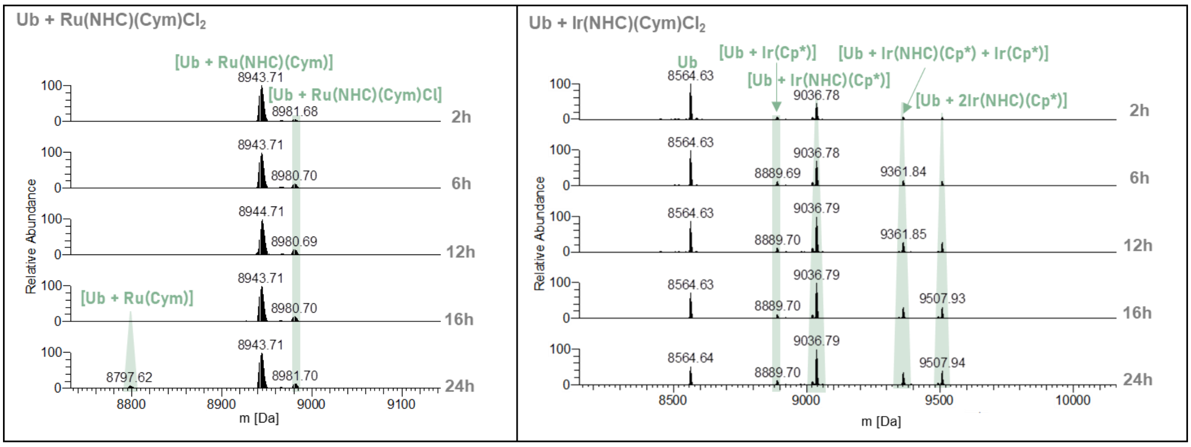

Figure S3. Time-resolved mass spectra of Ub with compound **4** (left) and compound **6** (right).

3) Complex and adduct speciation

Compound 6:

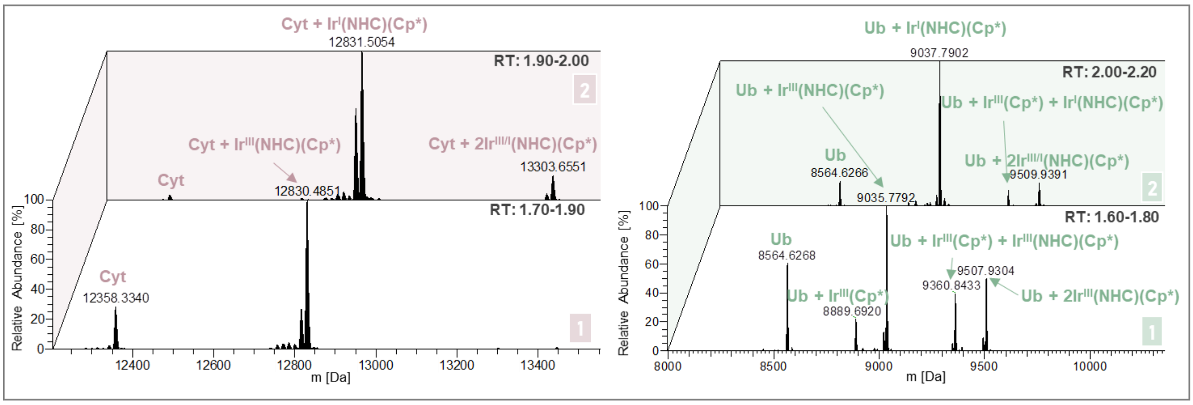

Figure S4. Deconvoluted mass spectra of the Cyt and Ub adducts with compound **6**.

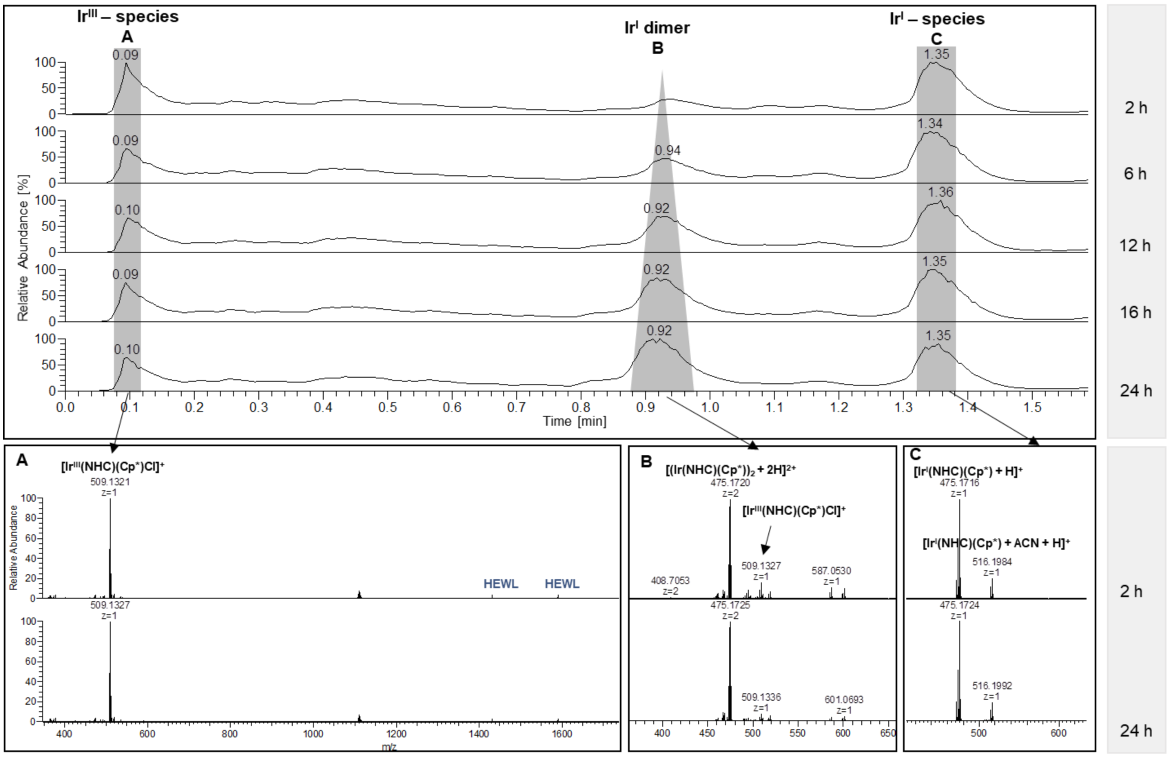

Figure S5. Top: TIC of the free Ir species of compound **6** after 2, 6, 12, 16, and 24 h incubation with the protein mixture. Bottom: Mass spectra of the Ir species of compound **6** after 2 and 24 h incubation with the protein mixture. Over time, a dimeric species formed (B) for which the oxidation state is unclear.

Table S4. Identified free complex species of compound **6** with sum formula, m/z, error and retention time (RT) in the chromatogram.

Spectrum	Species	Sum formula	m/z exp	m/z theo	Error /ppm	RT /min
**A + B**	$[Ir^{III}(NHC)(Cp^*)Cl]^+$	$Ir(C_{10}H_{15})(C_9H_{10}N_2)Cl$	509.1327	509.1321	1.18	0.10
**B**	$[(Ir(NHC)(Cp^*))_2 + 2H]^{2+}$	$(Ir(C_{10}H_{15})(C_9H_{10}N_2))_2H_2$	475.1725	475.1722	0.63	0.92
**C**	$[Ir^{I}(NHC)(Cp^*) + H]^+$	$Ir(C_{10}H_{15})(C_9H_{10}N_2)H$	475.1724	475.172	0.84	1.35
	$[Ir^{I}(NHC)(Cp^*) + ACN + H]^+$	$Ir(C_{10}H_{15})(C_9H_{10}N_2)HCH_3CN$	516.1991	516.1986	0.97	1.35

4) LC-MS/MS-fragmentation of selected precursor ions:

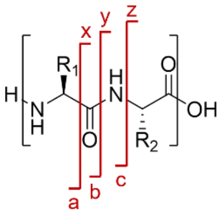

Figure S6. MS/MS fragment nomenclature of proteins.

Table S5. m/z values, mass errors, RT and signal intensity of the identified fragments (Cyt – red, Ub – green, HEWL – blue, Myo – ocre; precursor ions highlighted).

Complex	Fragments	Site	m/z_{exp} /-	m/z_{theo} /-	Error /ppm	NCE	Intensity /-
**1** $Pt(NH_3)_2Cl_2$	$[Cyt^*_{+14H} + Pt^{II}(NH_3)_2Cl^{(+1)}]^{15+}$	-	-	842.4951	-	20	-
	$[y39_{+6H}]^{6+}$	S_{Met65}	763.0913	763.0894	2.49	20	2.20E5
	$[y39_{+6H} - H_2O]^{6+}$	S_{Met65}	760.0901	760.0877	3.16	20	3.45E5
	$[y39_{+5H}]^{5+}$	S_{Met65}	915.5077	915.5059	1.97	20	3.23E5
	$[y29_{+4H}]^{4+}$	-	824.4660	824.4634	3.15	20	2.30E5
	$[b45^*_{+5H}]^{6+}$	-	903.1239	903.1215	2.66	20	1.64E5
	$[b46^*_{+5H}]^{6+}$	-	927.6343	927.6334	0.97	20	1.36E5
	$[b47^*_{+5H}]^{6+}$	-	944.4762	944.4745	1.80	20	9.35E4
	$[b47^*_{+6H}]^{7+}$	-	809.6958	809.6936	2.72	20	7.45E4
	$[y43_{+6H} + Pt^{II}Cl^{(+1)} - H_2O]^{6+}$	S_{Met65}	877.4456	877.4443	1.48	20	1.08E5
	$[y43_{+7H} + Pt^{II}Cl^{(+1)} - H_2O]^{7+}$	S_{Met65}	752.2398	752.2392	0.80	20	5.39E4
	$[a65^*_{+7H} + Pt^{(+2)}]^{9+}$	S_{Met65}	884.3205	884.3182	2.60	20	1.99E5
	$[a65^*_{+7H} + Pt^{(+2)} - H_2O]^{9+}$	S_{Met65}	882.3202	882.3164	4.31	20	1.11E5
	$[b65^*_{+7H} + Pt^{(+2)}]^{9+}$	S_{Met65}	887.4294	887.4282	1.35	20	9.73E4
	$[b65^*_{+8H} + Pt^{II}Cl^{(+1)}]^{9+}$	S_{Met65}	891.5396	891.5368	3.14	20	4.98E4
**2** Pt(ala)(ASA -But)Cl	$[Cyt^*_{+15H} + Pt^{II}(ala)Cl^{(0)}]^{15+}$	-	846.0974	846.0944	3.55	20	1.22E5
	$[y39_{+6H}]^{6+}$	S_{Met65}	763.0917	763.0894	3.01	20	3.29E5
	$[y39_{+6H} - H_2O]^{6+}$	S_{Met65}	760.0910	760.0877	4.34	20	6.20E5
	$[y39_{+5H}]^{5+}$	S_{Met65}	915.5078	915.5059	2.08	20	3.38E5
	$[y29_{+4H}]^{4+}$	-	824.4669	824.4634	4.25	20	2.25E5
	$[b45^*_{+6H}]^{6+}$	-	902.9567	902.9544	2.55	20	1.24E5
	$[b46^*_{+6H}]^{6+}$	-	927.6349	927.6329	2.16	20	6.61E6
	$[b47^*_{+6H}]^{6+}$	-	944.4763	944.4745	1.91	20	1.30E5
	$[b47^*_{+7H}]^{7+}$	-	809.6963	809.6932	3.83	20	1.01E5
	$[Cyt^*_{+15H} + Pt^{II}(ala)Cl^{(0)} - H_2O]^{15+}$	-	844.9629	844.9606	2.72	20	8.18E4
	$[Cyt^*_{+13H} + Pt^{(+2)}]^{15+}$	-	837.7618	837.7598	2.39	20	2.03E4
	$[y56_{+8H} + Pt(ala)Cl^{(0)}]^{8+}$	S_{Met65}	857.5643	857.5628	1.75	20	1.00E5
	$[y57_{+9H} + Pt(ala)Cl^{(0)}]^{9+}$	S_{Met65}	780.5091	780.5079	1.54	20	2.74E4
	$[a65^*_{+7H} + Pt^{(+2)}]^{9+}$	S_{Met65}	884.3206	884.3177	3.27	20	1.96E5
	$[a65^*_{+7H} + Pt^{(+2)} - H_2O]^{9+}$	S_{Met65}	882.3206	882.3165	4.65	20	8.29E4
	$[b56^*_{+7H} + Pt^{(+2)}]^{9+}$	S_{Met65}	887.4306	887.4282	2.70	20	1.02E5
	$[b56^*_{+8H} + Pt^{II}Cl^{(+1)}]^{9+}$	S_{Met65}	891.5393	891.5368	2.80	20	3.99E4
	$[Cyt^*_{+15H} + 2Pt^{II}(ala)Cl^{(0)}]^{15+}$	-	867.2923	867.2924	-0.12	20	2.54E4
	$[y24_{+3H}]^{3+}$	S_{Met80}	927.5306	927.5297	0.97	20	1.27E5
	$[y24_{+4H}]^{4+}$	S_{Met80}	695.9002	695.8988	2.01	20	9.27E4
	$[b45^*_{+6H}]^{6+}$	-	902.9552	902.9544	0.89	20	6.14E6
	$[b46^*_{+6H}]^{6+}$	-	927.6337	927.6334	0.32	20	6.78E4
	$[b47^*_{+6H}]^{6+}$	-	944.4755	944.4742	1.38	20	6.00E4
	$[b47^*_{+7H}]^{7+}$	-	809.6944	809.6936	0.99	20	2.51E4
	$[y39_{+5H} + Pt^{II}(ala)Cl^{(0)}]^{5+}$	S_{Met80}	979.3016	979.3006	1.02	20	3.01E4

	[y56$_{+8H}$ + 2Pt^{II}(ala)Cl$^{(0)}$]$^{8+}$	S$_{Met65}$, S$_{Met80}$	897.4351	897.4342	1.00	20	2.66E4
	[y86$_{+11H}$ + Pt^{II}(ala)Cl$^{(0)}$]$^{11+}$	-	911.7461	911.7444	1.86	20	3.60E4
	[a65*$_{+7H}$ + Pt$^{(+2)}$]$^{9+}$	S$_{Met65}$	884.3203	884.3177	2.94	20	1.96E5
	[a65*$_{+7H}$ + Pt$^{(+2)}$ - H_2O]$^{9+}$	S$_{Met65}$	882.3185	882.3165	2.27	20	2.47E4
	[b56*$_{+7H}$ + Pt$^{(+2)}$]$^{9+}$	S$_{Met65}$	887.4300	887.4282	2.03	20	2.46E4
	[b56*$_{+8H}$ + Pt^{II}Cl$^{(+1)}$]$^{9+}$	S$_{Met65}$	891.5375	891.5361	1.57	20	1.19E4
**4** Ru(NHC)(Cym)Cl_2	[Cyt*$_{+13H}$ + Ru^{II}(NHC)(Cym)$^{(2+)}$]$^{15+}$	-	850.1686	850.168	0.71	20	6.12E4
	[y29$_{+4H}$]$^{4+}$	-	824.464	824.4634	0.73	25	4.70E4
	[y29$_{+3H}$]$^{3+}$	-	1098.9488	1098.9488	0.00	25	3.59E4
	[y25$_{+2H}$]$^{3+}$	-	971.2099	971.2094	0.51	25	2.25E4
	[y24$_{+2H}$]$^{3+}$	-	927.5294	927.5292	0.22	25	2.00E4
	[y40$_{+4H}$]$^{5+}$	-	941.7142	941.7139	0.32	25	1.64E4
	[y22$_{+2H}$]$^{3+}$	-	840.8116	840.8121	-0.59	25	1.83E4
	[y56$_{+7H}$]$^{8+}$	-	817.8169	817.8161	0.98	20	6.12E4
	[y57$_{+7H}$]$^{8+}$	-	838.0741	838.0737	0.48	20	9.14E4
	[y58$_{+7H}$]$^{8+}$	-	850.7047	850.705	-0.35	20	7.04E4
	[b43*$_{+4H}$ + Ru(NHC)(Cym)$^{(+2)}$]$^{6+}$	N$_{His26/33}$	940.7907	940.7907	0.00	20	2.65E4
	[b46*$_{+4H}$ + Ru(NHC)(Cym)$^{(+2)}$]$^{6+}$	N$_{His26/33}$	990.9828	990.9817	1.11	20	1.96E4
	[b45*$_{+4H}$ + Ru(NHC)(Cym) $^{(+2)}$]$^{6+}$	N$_{His26/33}$	966.3026	966.3034	-0.83	20	1.91E4
	[b47*$_{+5H}$ + Ru(NHC)(Cym)$^{(+2)}$]$^{7+}$	N$_{His26/33}$	863.8488	863.8489	-0.12	20	1.79E4
**1** Pt$(NH_3)_2Cl_2$	[Ub$_{+8H}$ + Pt^{II}$(NH_3)_2$Cl$^{(+1)}$]$^{9+}$	-	981.8522	981.8521	0.10	20	1.04E5
	[Ub$_{+7H}$ + Pt$^{(+2)}$]$^{9+}$	-	974.0727	974.0716	1.13	20	4.60E5
	[Ub$_{+7H}$ + Pt^{II}$(NH_3)$$^{(+2)}$]$^{9+}$	-	975.8526	975.8520	0.61	20	1.94E5
	[Ub$_{+7H}$ + Pt^{II}$(NH_3)_2$$^{(+2)}$]$^{9+}$	-	977.7433	977.7438	-0.51	20	1.09E5
	[Ub$_{+8H}$ + Pt^{II}(NH_3)Cl$^{(+1)}$]$^{9+}$	-	979.9616	979.9607	0.92	20	1.91E5
	[y58$_{+4H}$ + Pt$^{(+2)}$]$^{6+}$	N$_{His68}$	1121.5836	1121.5816	1.69	25	4.60E4
	[y53$_{+4H}$ + Pt$^{(+2)}$]$^{6+}$	N$_{His68}$	1036.0430	1036.0412	1.74	25	1.81E4
	[y40$_{+3H}$ + Pt$^{(+2)}$]$^{5+}$	N$_{His68}$	952.2910	952.2885	2.63	25	9.01E3
	[y52$_{+4H}$ + Pt$^{(+2)}$]$^{6+}$	N$_{His68}$	1014.5361	1014.5339	2.17	25	7.49E3
	[y40$_{+2H}$ + Pt$^{(+2)}$]$^{4+}$	N$_{His68}$	1190.1108	1190.1088	1.68	25	6.43E3
**2** Pt(ala)(ASA-But)Cl	[Ub$_{+9H}$ + Pt^{II}(ala)$^{(+1)}$]$^{10+}$	-	885.6719	885.6699	2.26	20	1.90E6
	[y58$_{+7H}$]$^{7+}$	-	933.9383	933.9355	3.00	20	2.07E6
	[y58$_{+8H}$]$^{8+}$	-	817.3224	817.3193	3.79	20	1.28E6
	[y59 - $H_2O$$_{+7H}$]$^{7+}$	-	949.7995	949.7972	2.42	20	2.67E5
	[y59$_{+7H}$]$^{7+}$	-	952.3724	952.3701	2.42	20	2.55E5
	[y40$_{+5H}$]$^{5+}$	-	913.7023	913.6987	3.94	20	3.09E5
	[b2 + Pt^{II}(ala)$^{(+1)}$]$^{+}$	S$_{Met1}$	542.1047	542.1032	2.77	20	5.60E4
	[b14$_{+2H}$ + Pt^{II}(ala)$^{(+1)}$ - H_2O]$^{3+}$	S$_{Met1}$	609.9683	609.9664	3.11	20	5.56E4
	[a15$_{+2H}$ + Pt^{II}(ala)$^{(+1)}$]$^{3+}$	S$_{Met1}$	644.3359	644.3333	4.04	20	9.89E4
	[b15$_{+2H}$ + Pt^{II}(ala)$^{(+1)}$]$^{3+}$	S$_{Met1}$	653.6680	653.6650	4.59	20	8.33E4
	[b16$_{+2H}$ + Pt^{II}(ala)$^{(+1)}$]$^{3+}$	S$_{Met1}$	696.6816	696.6796	2.87	20	6.39E5
	[a17$_{+2H}$ + Pt^{II}(ala)$^{(+1)}$]$^{3+}$	S$_{Met1}$	720.3732	720.3706	3.61	20	1.21E5
	[b17$_{+2H}$ + Pt^{II}(ala)$^{(+1)}$]$^{3+}$	S$_{Met1}$	729.7043	729.7020	3.15	20	7.23E5
	[b18$_{+2H}$ + Pt^{II}(ala)$^{(+1)}$]$^{3+}$	S$_{Met1}$	772.7189	772.7162	3.49	20	9.37E5
**4** Ru(NHC)(Cym)Cl_2	[Ub$_{+8H}$ + Ru^{II}(NHC)(cym)$^{(+2)}$]$^{10+}$	-	895.4787	895.4787	0.00	25	2.33E3
	[y40$_{+5H}$]$^{5+}$	-	913.6993	913.6987	0.66	25	2.95E4
	[y40$_{+4H}$]$^{4+}$	-	1141.8718	1141.8711	0.61	25	3.60E4
	[y58$_{+6H}$]$^{6+}$	-	1089.4229	1089.4231	-0.18	25	2.11E4
	[y14$_{+2H}$]$^{2+}$	-	789.9756	789.9756	0.00	25	3.06E4
	[y13$_{+2H}$]$^{2+}$	-	725.9283	725.9281	0.28	25	3.14E4
	[y15$_{+2H}$]$^{2+}$	-	854.0049	854.0048	0.12	25	8.54E2
	[y24$_{+3H}$]$^{3+}$	-	909.8403	909.8411	-0.88	25	1.42E4
	[b16$_{+2H}$]$^{2+}$	-	903.0155	903.0157	-0.22	25	1.76E4
	[y12$_{+2H}$]$^{2+}$	-	661.4072	661.4068	0.60	25	1.89E4
	[y11$_{+2H}$]$^{2+}$	-	617.8911	617.8908	0.49	25	1.54E4
	[Ru^{II}(cym)(NHC)$^{(+2)}$$_{-H}$]$^{+}$	-	381.0897	381.0905	-2.10	25	7.06E4

	[y58$_{+5H}$ + Ru^{II}(cym)(NHC)$^{(+2)}$]$^{7+}$	N$_{His68}$	988.2342	988.2333	0.91	25	5.43E4
	[y58$_{+6H}$ + Ru^{II}(cym)(NHC)$^{(+2)}$]$^{8+}$	N$_{His68}$	864.8302	864.8298	0.46	25	1.70E4
	[y57$_{+5H}$ + Ru^{II}(cym)(NHC)$^{(+2)}$]$^{7+}$	N$_{His68}$	974.3693	974.3684	0.92	25	1.40E4
	[y40$_{+4H}$ + Ru^{II}(cym)(NHC)$^{(+2)}$]$^{6+}$	N$_{His68}$	824.9313	824.9309	0.48	25	1.26E4
	[y42$_{+3H}$ + Ru^{II}(cym)(NHC)$^{(+2)}$]$^{5+}$	N$_{His68}$	1023.7372	1023.7369	0.29	25	1.04E4
	[y37$_{+3H}$ + Ru^{II}(cym)(NHC)$^{(+2)}$]$^{5+}$	N$_{His68}$	927.8893	927.8887	0.65	25	9.56E3
	[y43$_{+3H}$ + Ru^{II}(cym)(NHC)$^{(+2)}$]$^{5+}$	N$_{His68}$	1049.5442	1049.5454	-1.14	25	8.85E3
	[y24$_{+2H}$ + Ru^{II}(cym)(NHC)$^{(+2)}$]$^{4+}$	N$_{His68}$	777.6541	777.6541	0.00	25	1.03E4
	[y56$_{+4H}$ + Ru^{II}(cym)(NHC)$^{(+2)}$]$^{7+}$	N$_{His68}$	961.9354	961.9354	0.00	25	6.99E3
	[y50$_{+3H}$ + Ru^{II}(cym)(NHC)$^{(+2)}$]$^{6+}$	N$_{His68}$	1010.2048	1010.2051	-0.30	25	9.59E3
**6** Ir(NHC)(Cp*)Cl$_2$	[Ub$_{+6H}$ + Ir^{III}(NHC)(Cp*)$^{(+2)}$ + Ir^{III}(Cp*)$^{(+2)}$]$^{10+}$	-	937.1917	937.1917	0.00	20	3.82E4
	[y75$_{+8H}$]$^{8+}$	-	1055.0786	1055.0807	-1.99	20	8.78E5
	[Ub$_{+7H}$ +Ir(Cp)$^{(+2)}$]$^{9+}$	-	988.7495	988.7510	-1.52	20	3.82E5
	[b16$_{+H}$ + Ir(Cp*)$^{(+2)}$]$^{3+}$	S$_{Met1}$	711.0341	711.0347	-0.84	20	2.01E4
	[b17$_{+H}$ + Ir(Cp*)$^{(+2)}$]$^{3+}$	S$_{Met1}$	744.0568	744.0573	-0.67	20	2.58E4
	[b18$_{+H}$ + Ir(Cp*)$^{(+2)}$]$^{3+}$	S$_{Met1}$	787.0714	787.0718	-0.51	20	3.71E4

* (+ Ac + heme)

*Compound **1**:*

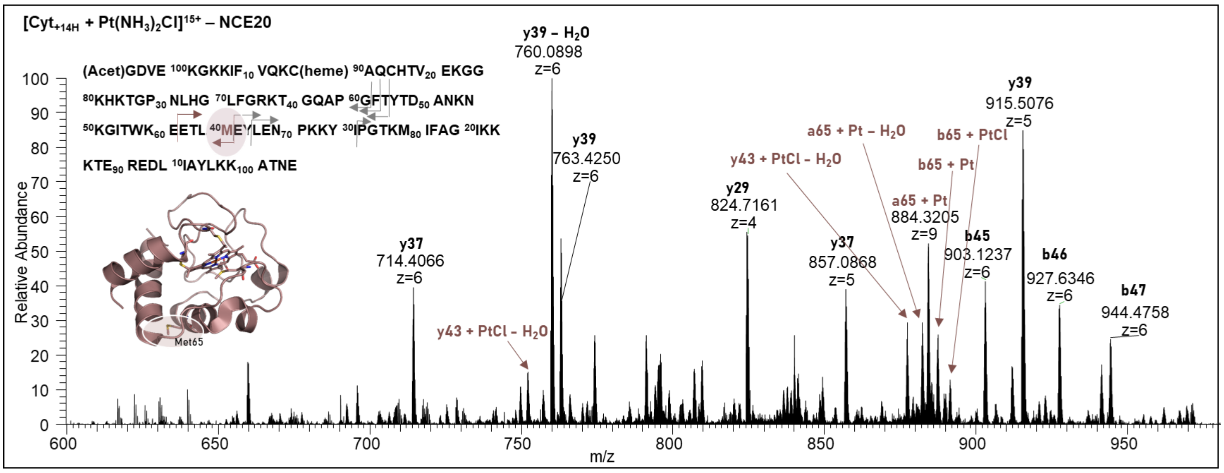

Figure S7. HCD fragmentation spectrum of [Cyt + Pt(NH$_3$)$_2$Cl] at NCE 20 with corresponding protein sequence and 3D structure of the protein (platination sites highlighted; metalated fragments are indicated in red and non-metalated fragments in grey).

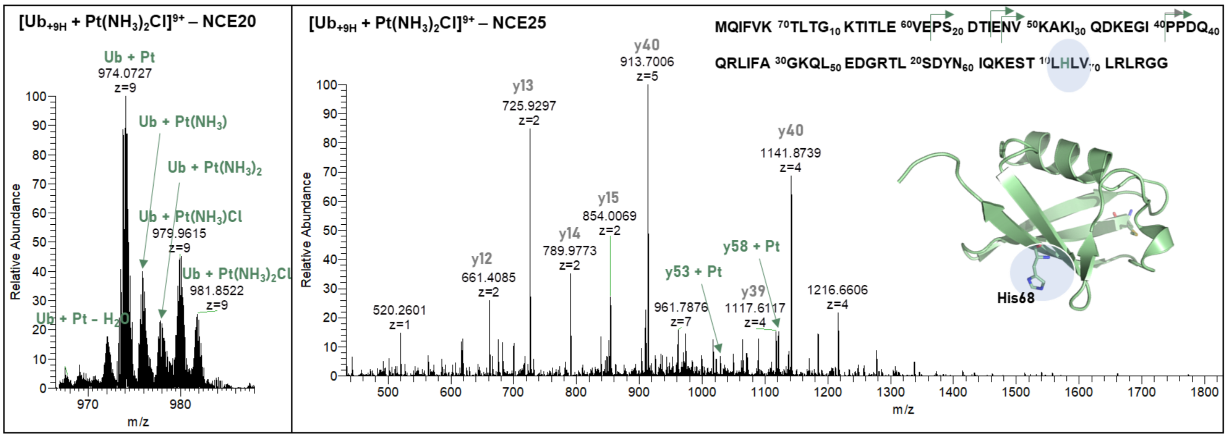

Figure S8. Left: HCD fragmentation spectrum of [Ub + Pt(NH$_3$)$_2$Cl] at NCE 20. Right: HCD fragmentation spectrum of [Ub + Pt(NH$_3$)$_2$Cl] at NCE 25 with corresponding protein sequence and 3D structure (platination sites highlighted; metalated fragments are indicated in green and non-metalated fragments in grey).

*Compound **4**:*

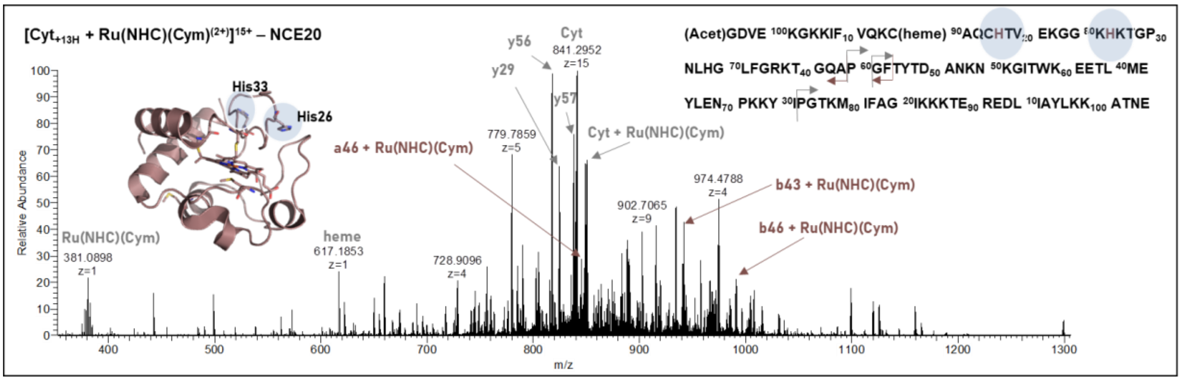

Figure S9: HCD fragmentation spectrum of [Cyt + Ru(NHC)(Cym)] at NCE 20 with corresponding protein sequence and 3D structure (platination sites highlighted; metalated fragments are indicated in red and non-metalated fragments in grey).

*Compound **6**:*

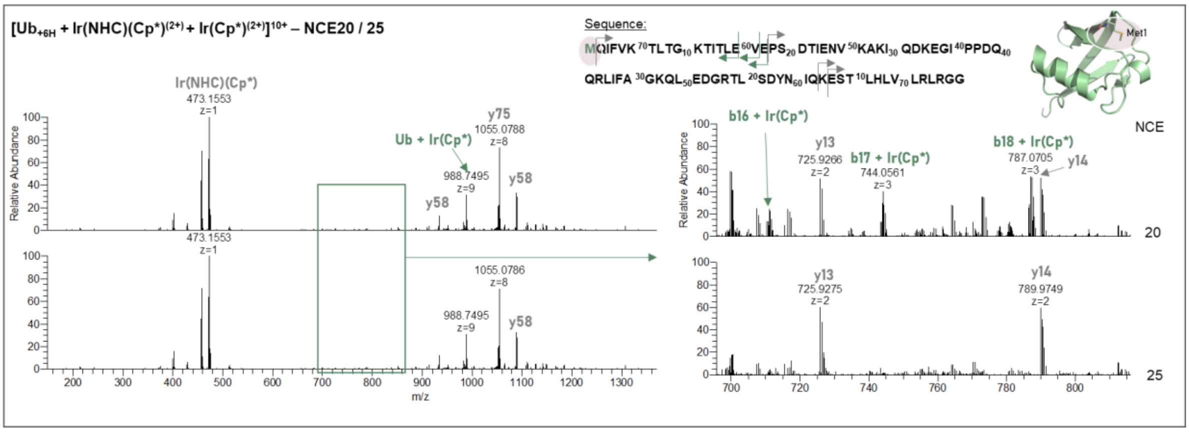

Figure S10: Left: HCD fragmentation spectra of [Ub + Ir(NHC)(Cp*) + Ir(Cp*)] at NCE 20 and 25. Right: Zoom into fragment spectra to show low abundant metalated fragments. Top: Protein Sequence with metalated fragments indicated in green and non-metalated fragments in grey; primary binding site for Ir(Cp*) highlighted.

*Compounds without metalated fragments in MS/MS Spectra (**3**, **5**, and **6**)*

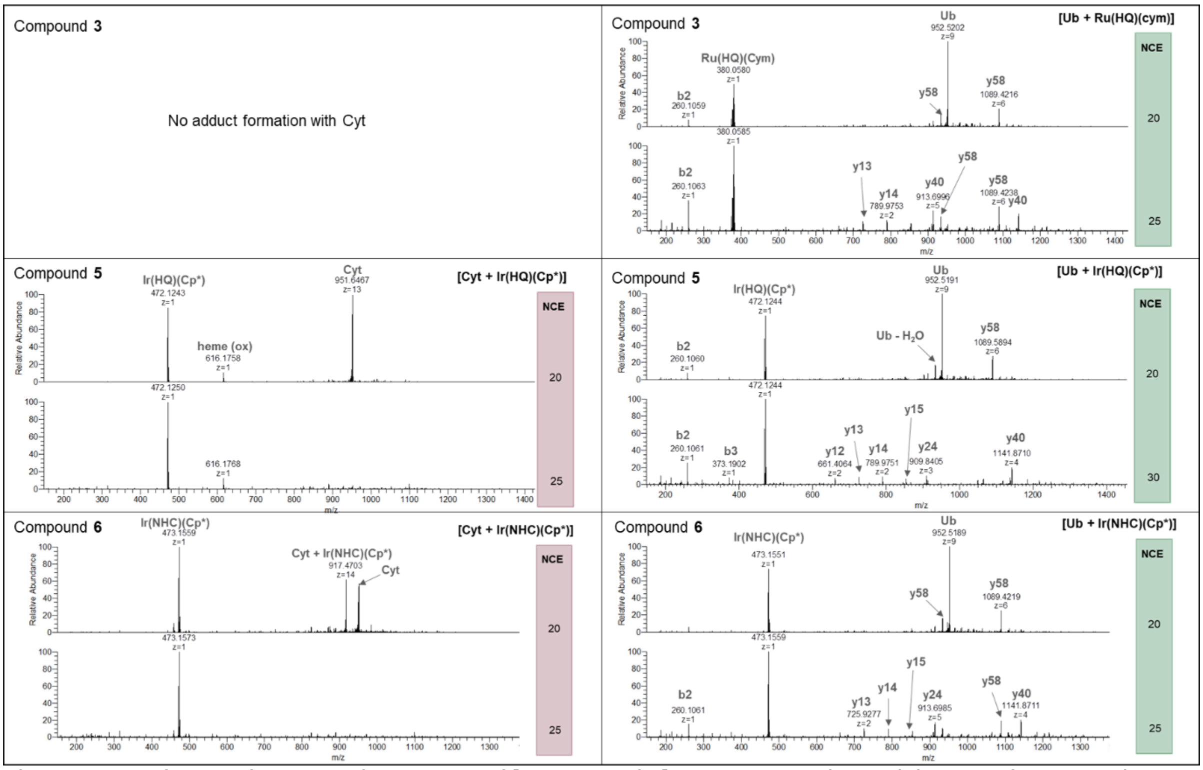

Figure S11. Left: HCD fragmentation spectra of [Cyt + **5** and **6**] at NCE 20 and 25. Right: HCD fragmentation spectra of [Ub + **3**, **5**, and **6**] at NCE 20 and 25 or 30.

5) Nucleophile exchange of compound 2 and 6:

Table S6. Adduct cleavage of compound **2** and **6** with Cyt and Ub after 0, 2, and 24 h nucleophile exposure with m/z values, mass errors, RT and signal intensity (Cyt – red, Ub – green).

Complex	Nuc exp. /h	Adduct	m/z_{exp} /-	m/z_{theo} /-	Error /ppm	Intensity /-
**2** Pt(ala)(ASA-But)Cl	0	**[Cyt*]**	**12358.3392**	**12358.3334**	**0.47**	**2.59E6**
		[Cyt*$_{-H}$ + PtCl$^{(1+)}$]	12588.2691	12588.2595	0.76	3.35E5
		[Cyt* + Pt(ala)Cl]	12677.3109	12677.3031	0.62	1.85E6
		[Cyt* + 2Pt(ala)Cl]	12995.2824	12995.2767	0.47	1.54E6
		[Cyt* + 3Pt(ala)Cl]	13314.2585	13314.2566	0.14	1.90E5
	2	**[Cyt*]**	**12358.3461**	**12358.3329**	**1.07**	**2.89E5**
		[Cyt*$_{-H}$ + PtCl$^{(1+)}$]	-	12588.2595	-	-
		[Cyt* + Pt(ala)Cl]	12677.3131	12677.3026	0.83	1.09E5
		[Cyt* + 2Pt(ala)Cl]	-	12995.2767	-	-
		[Cyt* + 3Pt(ala)Cl]	-	13314.2566	-	-
	24	**[Cyt*]**	**12358.3546**	**12358.3329**	**1.76**	**2.01E5**
		[Cyt*$_{-H}$ + PtCl$^{(1+)}$]	-	12588.2595	-	-
		[Cyt* + Pt(ala)Cl]	12677.3209	12677.3026	1.44	3.29E4
		[Cyt* + 2Pt(ala)Cl]	-	12995.2767	-	-
		[Cyt* + 3Pt(ala)Cl]	-	13314.2566	-	-
**6** Ir(NHC)(Cp*)Cl_2	0	**[Cyt*]**	**12358.3342**	**12358.3329**	**0.11**	**4.97E6**
		[Cyt*$_{-2H}$ + Ir(NHC)(Cp*)$^{(2+)}$]	12830.4872	12830.4855	0.13	7.05E6
		[Cyt*$_{-4H}$ + 2Ir(NHC)(Cp*)$^{(2+)}$]	13301.6424	13301.6296	0.96	2.26E5
	2	**[Cyt*]**	**12358.3272**	**12358.3385**	**-0.91**	**5.65E5**
		[Cyt*$_{-2H}$ + Ir(NHC)(Cp*)$^{(2+)}$]	12830.4808	12830.4855	-0.37	7.02E5
		[Cyt*$_{-4H}$ + 2Ir(NHC)(Cp*)$^{(2+)}$]	-	13301.6360	-	-

	24	**[Cyt*]**	**12358.3390**	**12358.3329**	**0.04**	**6.02E5**
		[Cyt*$_{-2H}$ + Ir(NHC)(Cp*)$^{(2+)}$]	12830.4919	12830.4855	0.50	3.77E5
		[Cyt*$_{-4H}$ + 2Ir(NHC)(Cp*)$^{(2+)}$]	-	13301.6360	-	-
**2** Pt(ala)(ASA-But)Cl	0	**[Ub]**	**8564.6244**	**8564.6308**	**-0.75**	**1.56E7**
		[Ub$_{-H}$ + Pt(ala)$^{(1+)}$]	8846.6232	8846.6265	-0.37	5.86E6
		[Ub + Pt(ala)Cl]	8882.6004	8882.6016	-0.14	8.52E5
	2	**[Ub]**	**8564.6250**	**8564.6302**	**-0.61**	**2.02E6**
		[Ub$_{-H}$ + Pt(ala)$^{(1+)}$]	8846.6254	8846.6212	0.47	5.57E5
		[Ub + Pt(ala)Cl]	-	889.2679	-	-
	24	**[Ub]**	**8564.6281**	**8564.6302**	**-0.25**	**2.12E6**
		[Ub$_{-H}$ + Pt(ala)$^{(1+)}$]	8846.6281	8846.6212	0.78	5.52E5
		[Ub + Pt(ala)Cl]	-	8882.6010	-	-
**6** Ir(NHC)(Cp*)Cl_2	0	**[Ub]**	**8564.6303**	**8564.6302**	**0.01**	**4.30E6**
		[Ub$_{-2H}$ + Ir(Cp*)$^{(2+)}$]	8889.6932	8889.6931	0.01	1.10E6
		[Ub$_{-2H}$ + Ir(NHC)(Cp*)$^{(2+)}$]	9035.7822	9035.7772	0.55	5.55E6
		[Ub$_{-4H}$ + Ir(NHC)(Cp*)$^{(2+)}$ + Ir(Cp*)$^{(2+)}$]	9361.8457	9361.8378	0.84	1.65E6
		[Ub$_{-4H}$ + 2Ir(NHC)(Cp*)$^{(2+)}$]	9506.9283	9506.9198	0.89	2.01E6
	2	**[Ub]**	**8564.6205**	**8564.6250**	**-0.53**	**6.10E5**
		[Ub$_{-2H}$ + Ir(Cp*)$^{(2+)}$]	8889.6819	8889.6931	-1.26	2.55E5
		[Ub$_{-2H}$ + Ir(NHC)(Cp*)$^{(2+)}$]	9035.7721	9035.7810	-0.98	9.75E5
		[Ub$_{-4H}$ + Ir(NHC)(Cp*)$^{(2+)}$ + Ir(Cp*)$^{(2+)}$]	9361.8361	9361.8438	-0.82	2.97E5
		[Ub$_{-4H}$ + 2Ir(NHC)(Cp*)$^{(2+)}$]	9507.9201	9507.9274	-0.77	3.50E5
	24	**[Ub]**	**8564.6261**	**8564.6250**	**0.13**	**1.40E6**
		[Ub$_{-2H}$ + Ir(Cp*)$^{(2+)}$]	8889.6943	8889.6931	0.13	4.29E5
		[Ub$_{-2H}$ + Ir(NHC)(Cp*)$^{(2+)}$]	9035.7781	9035.7810	-0.32	8.39E5
		[Ub$_{-4H}$ + Ir(NHC)(Cp*)$^{(2+)}$ + Ir(Cp*)$^{(2+)}$]	9361.8438	9361.8438	0.00	1.24E5
		[Ub$_{-4H}$ + 2Ir(NHC)(Cp*)$^{(2+)}$]	9507.9314	9507.9274	0.42	9.91E4

* (+ Ac + heme)

6) Nucleophile adducts of compounds 1 – 6:

Table S7. Identified nucleophile adducts with the compounds (m/z -values, mass errors, RT and signal intensity.)

Complex	Adduct	m/z_{exp} /-	m/z_{theo} /-	Error /ppm	RT /min	Intensity /-
**3** Ru(HQ)(cym)Cl	[Ru^{II}(HQ)(cym)]$^{+}$	380.0576	380.0588	-3.16	0.24	1.80E7
	[GS$^{(-1)}$$_{+H}$ + Ru^{II}(HQ)(cym)$^{(+1)}$ + 2O]$^{+}$	719.1308	719.1319	-1.53	0.21	7.11E4
**4** Ru(NHC)(cym)Cl_2	[Ru^{II} (NHC)(cym)$^{(+2)}$ - H]$^{+}$	381.0890	381.0905	-3.94	0.11	2.27E6
	[GS$^{(-1)}$$_{+H}$ + Ru^{II}(NHC)(cym)$^{(+2)}$]$^{+}$	688.1725	688.1748	-3.34	0.20	2.00E4
**5** Ir(HQ)(Cp*)Cl	[Ir^{III}(HQ)(Cp*)]$^{+}$	472.1242	472.1247	-1.06	0.29	5.93E7
	[GS$^{(-1)}$ + Ir^{III}(Cp*)$^{(+2)}$]$^{+}$	634.1552	634.1557	-1.58	0.09	2.34E6
	[2(GS$^{(-1)}$ + Ir^{III}(Cp*)$^{(+2)}$)]$^{2+}$	634.1576	634.1557	3.00	0.65	1.10E5
**6** Ir(NHC)(Cp*)Cl_2	[Ir^{III}(NHC)(Cp*)$^{(+2)}$$_{-H}$]$^{+}$	473.1567	473.1565	0.42	3.08	8.14E5
	[GS$^{(-1)}$$_{+H}$ + Ir^{III}(NHC)(Cp*)$^{(+2)}$]$^{2+}$	390.6230	390.6237	-1.79	0.22	1.23E8
	[GS$^{(-1)}$ + Ir^{III}(NHC)(Cp*)$^{(+2)}$]$^{+}$	780.2386	780.2401	-1.92	0.22	4.38E7
	[GS$^{(-1)}$ + Ir^{III}(NHC)(Cp*)$^{(+2)}$]$^{+}$$_{Na}$	802.2206	802.2220	-1.75	0.22	1.92E7
	[GS$^{(-1)}$ + Ir^{III}(NHC)(Cp*)$^{(+2)}$]$^{+}$$_{2Na}$	824.2025	824.2041	-1.94	0.22	3.17E6
	[GS$^{(-1)}$$_{-2H}$ + 2Ir^{III}(NHC)(Cp*)$^{(+2)}$]$^{+}$	1252.3907	1252.3895	0.96	1.28	9.15E4
	[GS$^{(-1)}$$_{-H}$ + 2Ir^{III}(NHC)(Cp*)$^{(+2)}$]$^{2+}$	625.6977	625.6970	1.12	1.28	3.52E6
	[GS$^{(-1)}$$_{-H}$ + 2Ir^{III}(NHC)(Cp*)$^{(+2)}$]$^{2+}$$_{Na}$	636.6881	636.6881	0.00	1.28	8.46E5
	[Met$^{(-1)}$$_{-H}$ + Ir(NHC)(Cp*)$^{(+2)}$]$^{+}$	622.2065	622.2073	-1.29	0.11	2.56E7

*Ru compounds (**3** + **4**):*

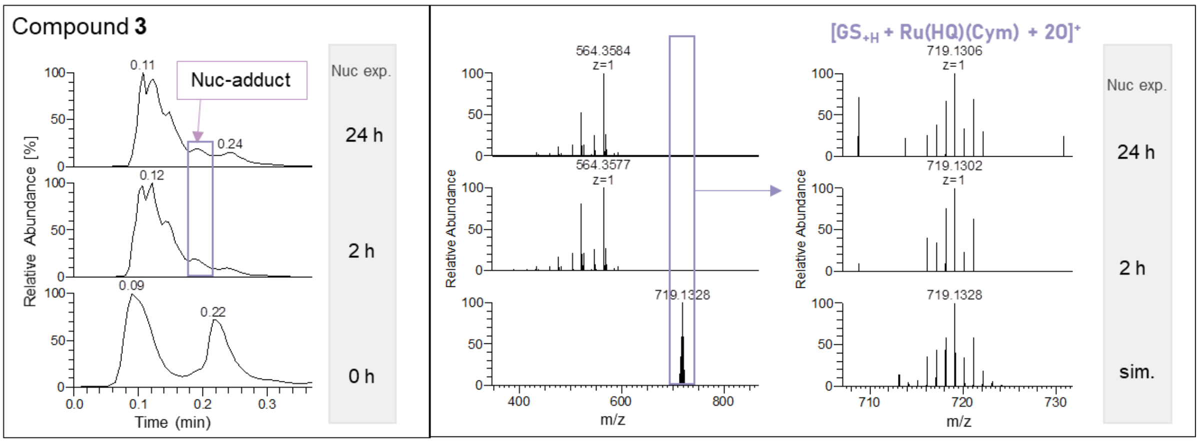

Figure S12. Nucleophile adduct with compound **3**. Left: TIC of the incubation mixture with compound **3** after 0, 2, and 24 h nucleophile exposure. Right: mass spectrum at RT = 0.19 min (nucleophile adduct elution) and comparison of experimental and simulated isotopic pattern.

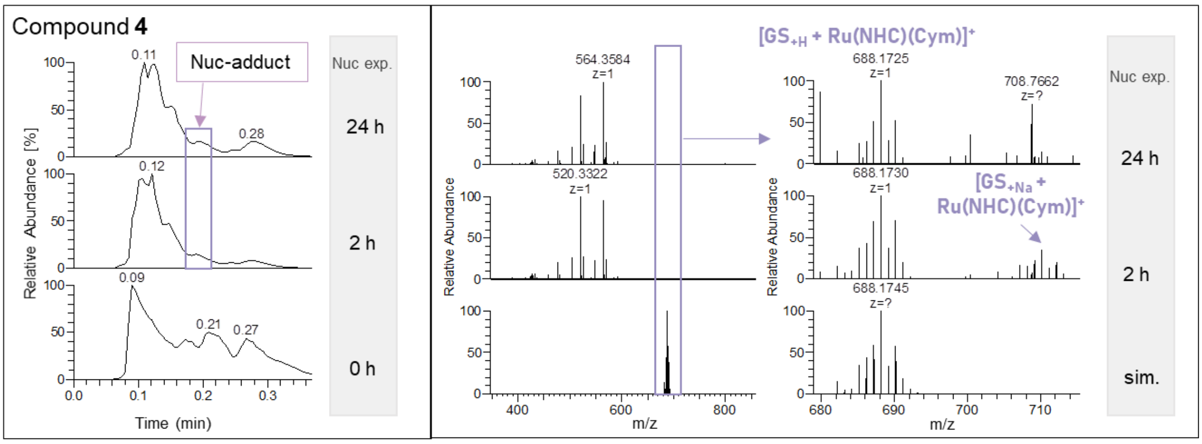

Figure S13. Nucleophile adduct with compound **4**. Left: TIC of the incubation mixture with compound **4** after 0, 2, and 24 h nucleophile exposure. Right: mass spectrum at RT = 0.19 min (nucleophile adduct elution) and comparison of experimental and simulated isotopic pattern.

*Ir Compound (**5**):*

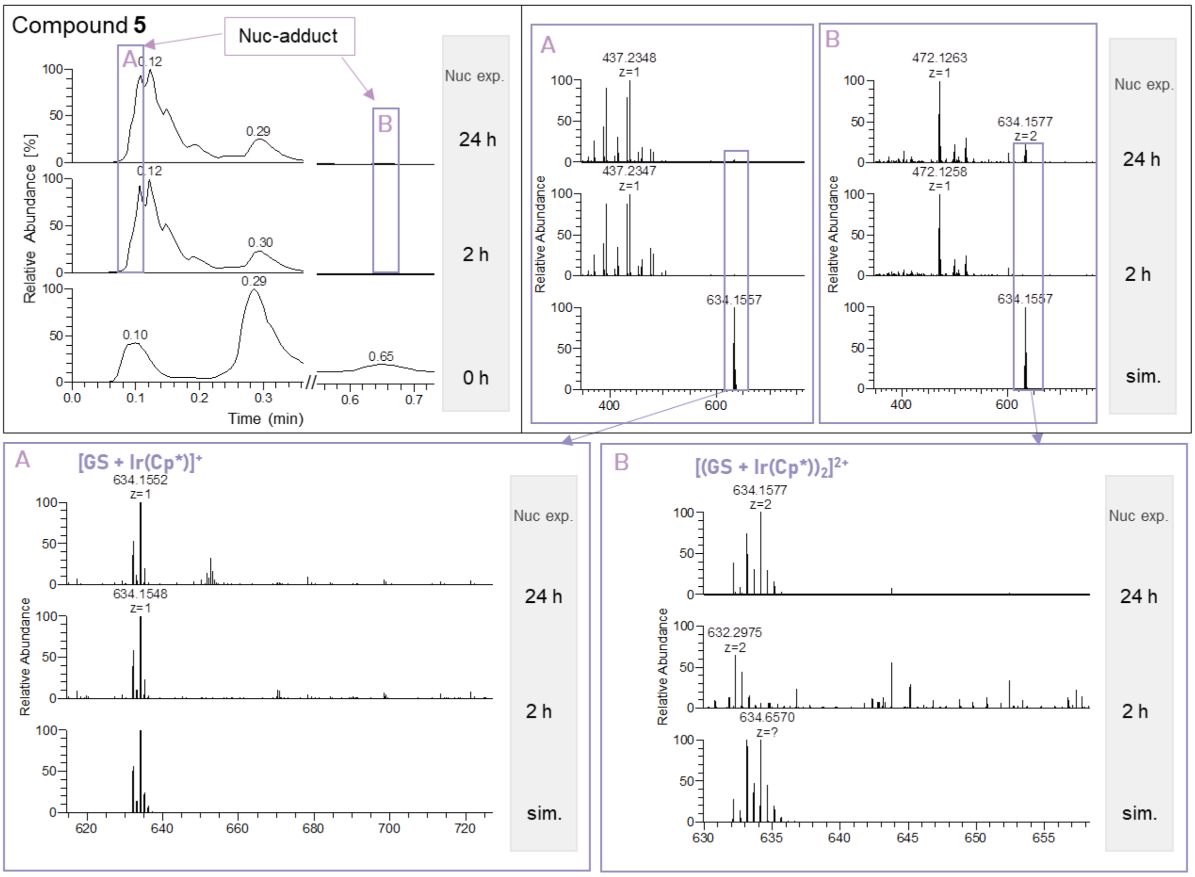

Figure S14. Nucleophile adduct with compound **5**. Top, left: TIC of the incubation mixture with compound **5** after 0, 2, and 24 h nucleophile exposure. Top, right: mass spectra at RT = 0.09 min and 0.65 min (nucleophile adduct elution). Bottom: comparison of experimental and simulated isotopic patterns.

6) Experiments in buffered solution

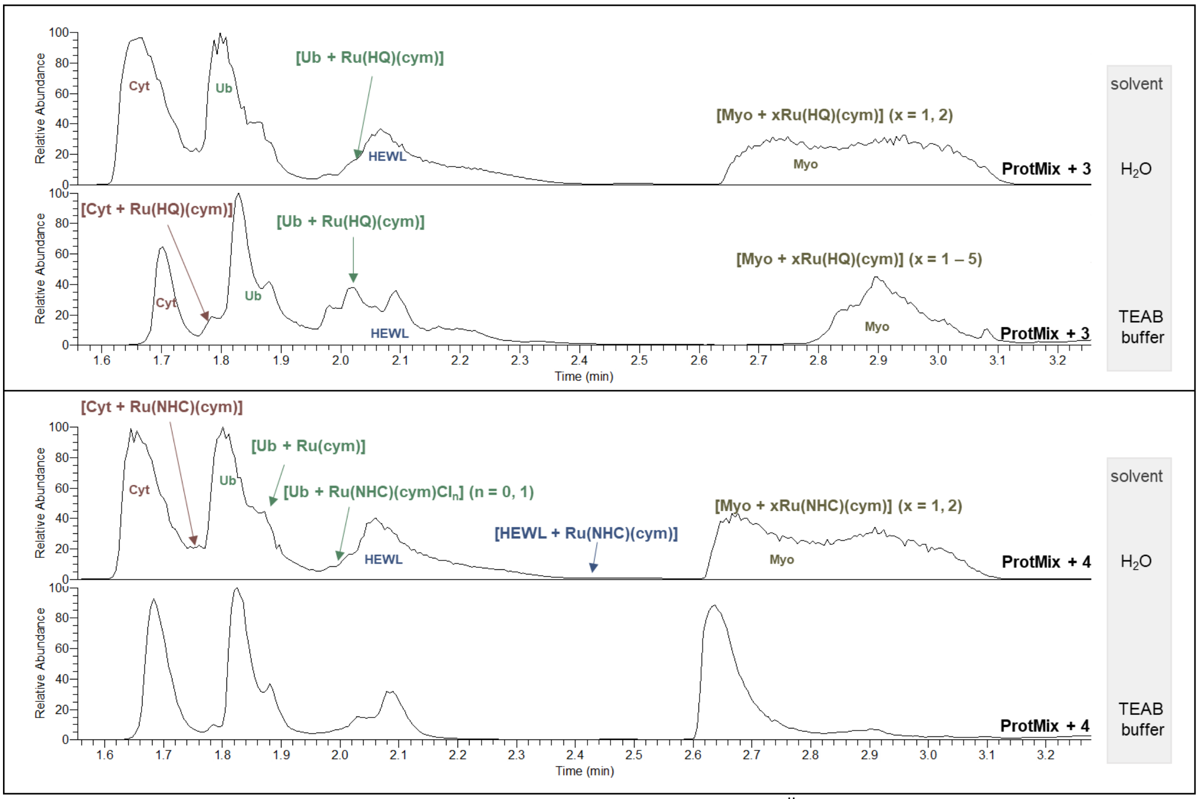

Figure S15. TICs after 24 h incubation of the protein mixtures with **3** – [Ru^{II}(HQ)(cym)Cl] and **4** – [Ru^{II}(NHC)(cym)Cl$_2$] in aqueous solution and 20 mM tetraethylammonium bicarbonate.

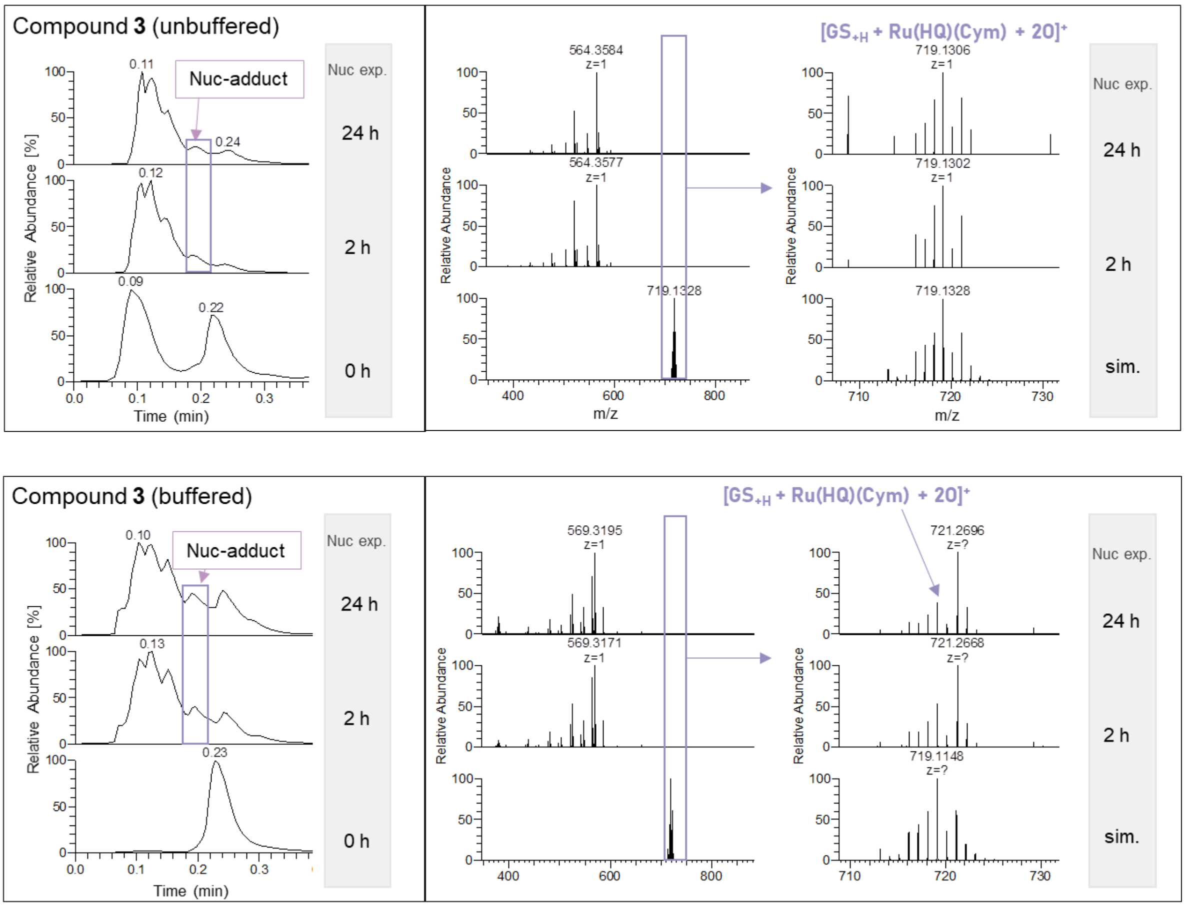

Figure S16. Comparison of the nucleophile adduct with compound **3** formed in aqueous solution (top) and 20 mM tetraethylammonium bicarbonate (bottom).
